# The normal development of *Platynereis dumerilii *(Nereididae, Annelida)

**DOI:** 10.1186/1742-9994-7-31

**Published:** 2010-12-30

**Authors:** Antje HL Fischer, Thorsten Henrich, Detlev Arendt

**Affiliations:** 1 Developmental Biology Unit, European Molecular Biology Laboratory, D-69117 Heidelberg, Germany; 2 International College, Osaka University, A217 School of Science Main Building 1-1, Machikaneyama-machi, Toyonaka, Osaka, 560-0043, Japan

## Abstract

**Background:**

The polychaete annelid *Platynereis dumerilii *is an emerging model organism for the study of molecular developmental processes, evolution, neurobiology and marine biology. Annelids belong to the Lophotrochozoa, the so far understudied third major branch of bilaterian animals besides deuterostomes and ecdysozoans. *P. dumerilii *has proven highly relevant to explore ancient bilaterian conditions via comparison to the deuterostomes, because it has accumulated less evolutionary change than conventional ecdysozoan models. Previous staging was mainly referring to hours post fertilization but did not allow matching stages between studies performed at (even slightly) different temperatures. To overcome this, and to provide a first comprehensive description of *P. dumerilii *normal development, a temperature-independent staging system is needed.

**Results:**

*Platynereis dumerilii *normal development is subdivided into 16 stages, starting with the zygote and ending with the death of the mature worms after delivering their gametes. The stages described can be easily identified by conventional light microscopy or even by dissecting scope. Developmental landmarks such as the beginning of phototaxis, the visibility of the stomodeal opening and of the chaetae, the first occurrence of the ciliary bands, the formation of the parapodia, the extension of antennae and cirri, the onset of feeding and other characteristics are used to define different developmental stages. The morphology of all larval stages as well as of juveniles and adults is documented by light microscopy. We also provide an overview of important steps in the development of the nervous system and of the musculature, using fluorescent labeling techniques and confocal laser-scanning microscopy. Timing of each developmental stage refers to hours post fertilization at 18 ± 0.1°C. For comparison, we determined the pace of development of larvae raised at 14°C, 16°C, 20°C, 25°C, 28°C and 30°C. A staging ontology representing the comprehensive list of developmental stages of *P. dumerilii *is available online.

**Conclusions:**

Our atlas of *Platynereis dumerilii *normal development represents an important resource for the growing *Platynereis *community and can also be applied to other nereidid annelids.

## Background

In the past decades, the annelid *Platynereis dumerilii *has been established as a marine animal model for developmental, evolutionary and neurobiological research as well as for ecology and toxicology [1-6]. It is especially suitable for comparative studies because several lines of evidence indicate that its evolutionary lineage has been slow-evolving. For example, *P. dumerilii *has a highly conserved gene structure [7] and genes involved in the development of the central nervous system are expressed in a conserved molecular topography in *P. dumerilii *and vertebrates [8,9]. Gene expression during development of the two-celled larval eye may reflect the bilaterian ground pattern [10].

Bilaterian animals comprise three main taxa: deuterostomes (e.g. chordates, hemichordates, echinoderms), ecdysozoans (e.g. arthropods, nematodes), and lophotrochozoans (mollusks, annelids and other marine invertebrates). "Classical", well-established animal models belong to the ecdysozoans (fruit fly, *C. elegans*) or deuterostomes (mouse, chicken, fish). Lophotrochozoans are still largely under-represented despite their obvious relevance to comparative approaches that seek to unravel the ground pattern of all bilaterians.

*P. dumerilii*, which has been kept in laboratory culture since 1953, easily breeds in captivity where it produces offspring throughout the year [6,11]. One single batch can contain more than 2000 eggs, which undergo embryonic and larval development in a highly synchronized manner [6]. Eggs, embryos and larvae are transparent and measure only 160 μm in diameter, making them accessible by conventional light microscopy as well as confocal laser-scanning microscopy (CLSM) in which structures throughout the organism can be visualized in whole mounts. They are well-suited for immunohistochemistry [12] and whole-mount *in situ *hybridization (WMISH) [13], which can be combined with confocal reflection microscopy [14], fluorescent WMISH and double WMISH [15]. Efficient microinjection techniques have paved the way for morpholino knock-down, RNAi and transgenesis (Arendt lab, unpublished data). Various transcriptomic and genomic resources have been generated and the whole genome has been sequenced (Arendt lab and others; unpublished).

Since the 1970 s, several studies have described different aspects of *P. dumerilii *development. Fischer [16,17] showed that the oocytes first develop asynchronously in clusters, connected by cytoplasmic bridges. Later during maturation, oocyte development becomes synchronized, the cytoplasmic bridges disappear and all the gametocytes of the female become mature and fertilizable synchronously [16,17]. The early phase of embryonic development was characterized by Dorresteijn *et al. *[18], Dorresteijn [5], Dorresteijn and Eich [19] and Ackermann *et al. *[20]. These studies revealed the specific contributions of individual blastomeres to the larval body. Also it was found that the fate of each blastomere is dependent upon the different amounts of nuclear β-catenin protein that results from asymmetric cell division during early embryogenesis [21]. Beginning with the eight-cell-stage, β-catenin shows a sister-cell asymmetry along the animal-vegetal axis following all cell divisions. Experimental ectopic activation of nuclear β-catenin leads to the adoption of the sister-cell fate [21]. In addition, the regional medio-lateral patterning and differentiation of the nervous system [8,9] and the development of the larval and adult eyes [2,10,22] have been investigated in greater detail. Morphometry revealed convergent extension movements in the neuroectoderm [23]. Segmentation, mushroom body development, mesoderm formation and the germ line development have also been studied [24-33].

### The terminology of *P. dumerilii *larval development

Polychaete larval development comprises traditionally three major stages: the trochophore, the metatrochophore and the nectochaete. The trochophore is a spherical larva characterized by an equatorial ciliated belt - the prototroch [34], and an apical organ with a ciliary tuft [34,35]. (Häcker [36] described an even earlier stage, called protrochophore, a pre-larva with a broad preoral band of short cilia but without mouth and anus.) The transition to the metatrochophore is accompanied by the development of a segmented trunk, which is slightly elongate in comparison to that of the trochophore [36]. The next stage is the nectochaete larva, following Häcker's [36] definition. The nectochaete larva bears parapodial appendages used for swimming and crawling, and resembles the adult in major traits.

This basic subdivision in trochophore, metatrochophore and nectochaete has also been applied to *P. dumerilii *[6,37].

An additional staging system currently used for *P. dumerilii *development refers to hours post fertilization (hpf) at 18.0°C [12]. This system allows for precise staging provided the temperature is kept constant. Given that the developmental rate of *P. dumerilii *is highly stereotyped between batches [5,6], the time-based system is sufficient for precise references. Furthermore, Fischer [38,39] introduced a staging system based on the morphology of the larval and adult eyes at 19°C ± 1°C. However, since minor temperature fluctuations cause significant changes in the pace of development, resulting differences in developmental speed complicate the comparison between studies. It is, for example, impossible to stage-match results from publications with 20°C [1] and 18°C reference temperature. This problem was also noted by Fischer [38,39].

In order to complement the hpf-based staging system with a temperature-independent reference system we define here a series of developmental stages, based on characters that are easily scored by conventional dissection or light microscopes and allow the comparison with other annelids. This is complemented by an overview of the development of the nervous system and body musculature, which offer additional diagnostic features for staging after antibody staining. The aim is to provide the growing community working on this annelid with a more refined, morphology-based reference system for *P. dumerilii *development and to present a synopsis with high temporal resolution of changes of morphological characters during postembryonic and larval development.

## Results

**Figure 1:**
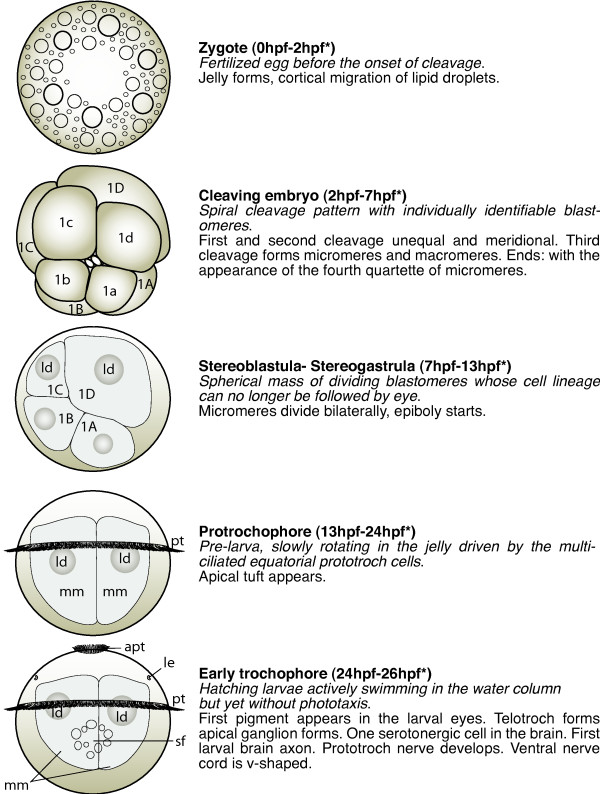
**Schemes of developmental stages of *P. dumerilii*: zygote, cleaving embryo, stereoblastula - stereogastrula, protrochophore and early trochophore**. Left: The scheme indicates the key characteristics of each developmental stage. Right: Next to the scheme a brief summary of the key features for the stage is given. The time points indicated with a star mark the end of each stage and are excluded from this stage. Bold: The name of the stage and in brackets the beginning and end of each stage is given. Italic: The key characteristics, which can be used to determine the beginning of each stage. Normal: Additional features of each stage are given, including some landmarks, which can be seen in the developing nervous system and musculature. From top to bottom: zygote (apical view), cleaving embryo (apical view), stereoblastula - stereogastrula (ventral view, apical up), protrochophore (ventral view, apical up) and early trochophore (ventral view, apical up). Abbreviations see abbreviations list.

**Figure 2:**
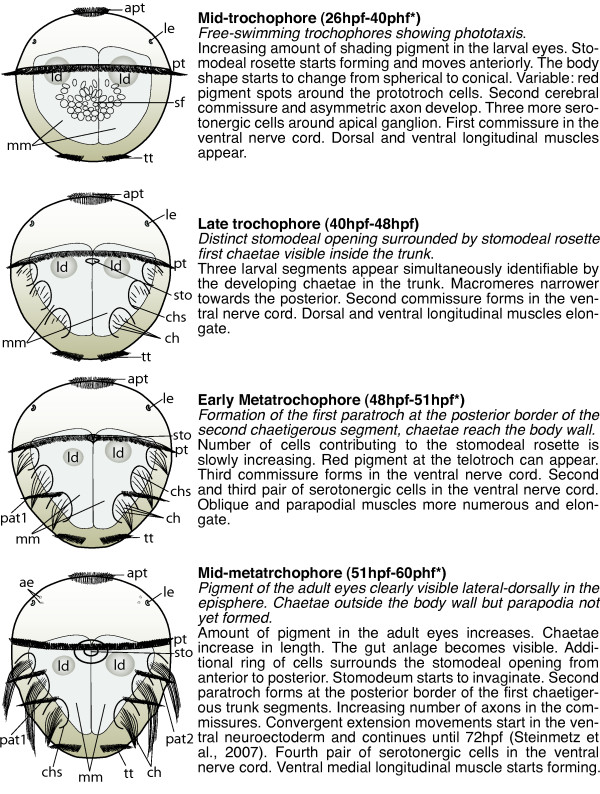
**Schemes of developmental stages of *P. dumerilii*: mid-trochophore, late trochophore, early metatrochophore and mid-metatrochophore**. Left: The scheme indicates the key characteristics of each developmental stage. Right: Next to the scheme a brief summary of the key features for the stage is given. The time points indicated with a star mark the end of each stage and are excluded from this stage. Bold: The name of the stage and in brackets the beginning and end of each stage is given. Italic: The key characteristics, which can be used to determine the beginning of each stage. Normal: Additional features of each stage are given, including some landmarks, which can be seen in the developing nervous system and musculature. From top to bottom: mid-trochophore, late trochophore, early metatrochophore, mid-metatrochophore. All schemes shown as ventral view, anterior up. Abbreviations see abbreviations list.

**Figure 3:**
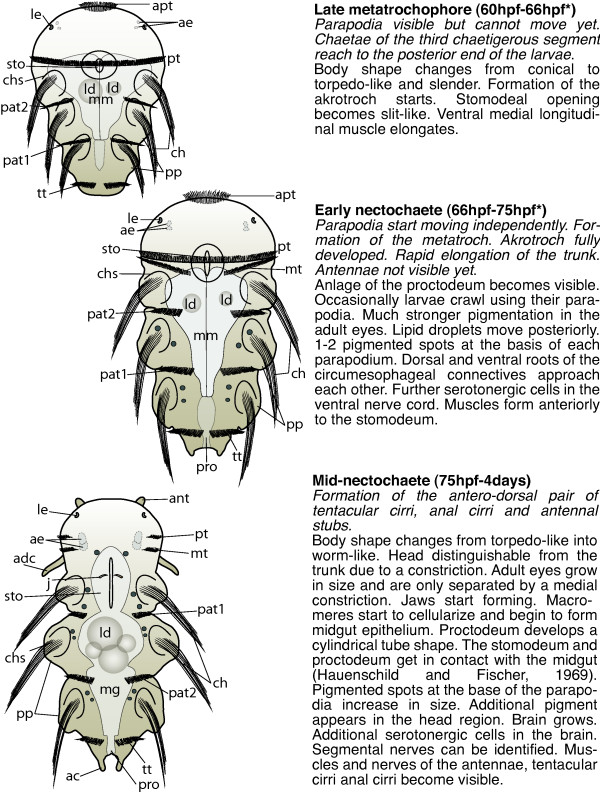
**Schemes of developmental stages of *P. dumerilii*: late metatrochophore, early nectochaete and mid-nectochaete**. Left: The scheme indicates the key characteristics of each developmental stage. Right: Next to the scheme a brief summary of the key features for the stage is given. The time points indicated with a star mark the end of each stage and are excluded from this stage. Bold: The name of the stage and in brackets the beginning and end of each stage is given. Italic: The key characteristics, which can be used to determine the beginning of each stage. Normal: Additional features of each stage are given, including some landmarks, which can be seen in the developing nervous system and musculature. From top to bottom: late metatrochophore, early nectochaete and mid-nectochaete. All schemes shown as ventral view, anterior left. Abbreviations see abbreviations list.

**Figure 4:**
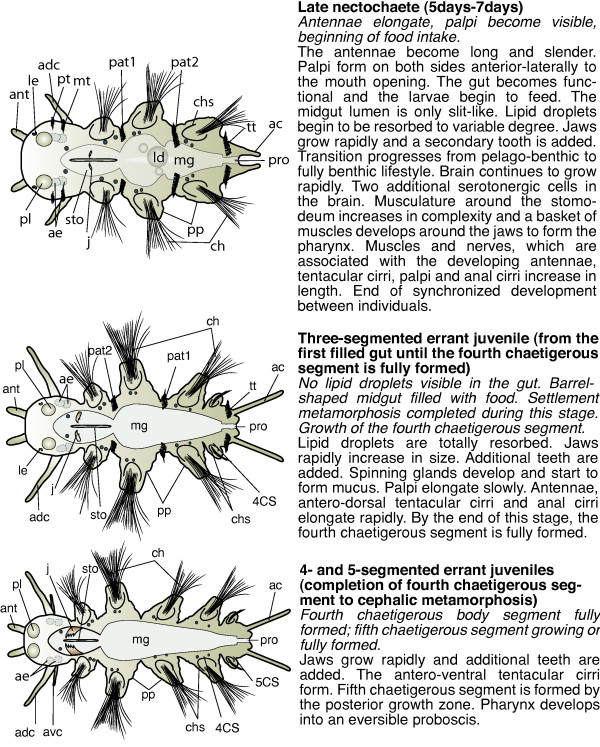
**Schemes of developmental stages of *P. dumerilii*: late nectochaete, three-segmented errant juvenile and 4- and 5-segmented errant juveniles**. Left: The scheme indicates the key characteristics of each developmental stage. Right: Next to the scheme a brief summary of the key features for the stage is given. Bold: The name of the stage and in brackets the beginning and end of each stage is given. Italic: The key characteristics, which can be used to determine the beginning of each stage. Normal: Additional features of each stage are given. From top to bottom: late nectochaete, three-segmented errant juvenile, Four- and five-segmented errant juvenile. All schemes shown as ventral view, anterior left. Abbreviations see abbreviations list.

**Figure 5:**
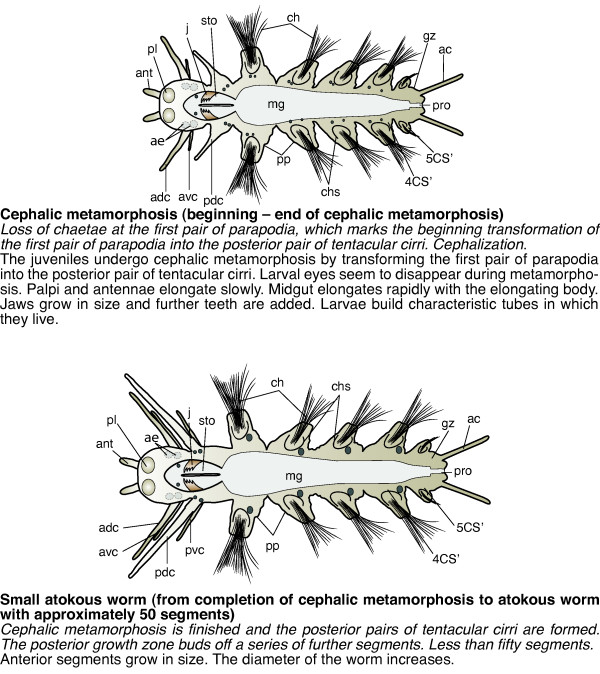
**Schemes of developmental stages of *P. dumerilii*: cephalic metamorphosis and small atokous worm**. Top: The scheme indicates the key characteristics of each developmental stage. Below each scheme: A brief summary of the key features for the stage is given. Bold: The name of the stage and in brackets the beginning and end of each stage is given. Italic: The key characteristics, which can be used to determine the beginning of each stage. Normal: Additional features of each stage are given. From top to bottom: cephalic metamorphosis, small atokous worm. All schemes shown as ventral view, anterior left. Abbreviations see abbreviations list.

**Figure 6:**
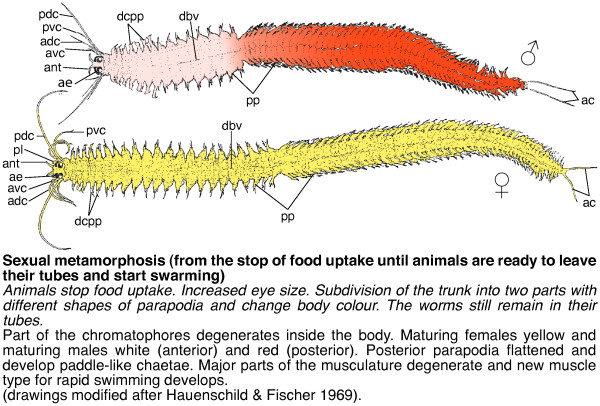
**Schemes of developmental stages of *P. dumerilii*: Sexual metamorphosis: male and female/heteronereis**. Top: The scheme indicates the key characteristics of the mature male and female heteronereis. Bottom: A brief summary of the key features for this stage is given. Bold: The name of the stage and in brackets the beginning and end of the stage is given. Italic: The key characteristics, which can be used to determine the beginning of each stage. Normal: Additional features of each stage are given. Schemes are shown as dorsal view, anterior left (schemes are modified from: [37]). Abbreviations see abbreviations list.

An overview of all developmental stages is given in the schemes in Figures 1, 2, 3, 4, 5 and 6.

### Embryonic stages

Embryonic development from the fertilized egg into a protrochophore. Cleavages, transition from spiralian type to bilaterally symmetrical cell divisions, determination of body axis and germ layer formation. No active locomotion. Protecting jelly mass, which enhances floating, surrounds embryos.

### Zygote (0 hpf-2 hpf)

Diagnostic feature: fertilized egg before the onset of cleavage (scheme: Figure 1).

The zygotes of *P. dumerilii *are around 160 μm in diameter [5] and slightly ellipsoidal in shape. The morphological changes of the zygote after fertilization have been described [5] and are therefore only summarized here. The early zygote contains protein yolk granules, cortical granules and numerous lipid droplets, which are distributed in the yolk in a highly organized manner. The cortical reaction, which is a complex change of the egg surface and a discharge of cortical granules upon fertilization leads to the formation of the egg jelly [40,41]. In culture, the simultaneous appearance of the jelly around all fertilized eggs in a batch forces them into a matrix, or honeycomb-like arrangement at the bottom of the cup indicating successful fertilization. Expansion of the jelly mass stops after approximately 40 min and only then can the jelly be removed, allowing the eggs to be treated further, if necessary. After the two polar bodies are formed, yolk granules migrate towards the vegetal pole and clear cytoplasm from the center of the egg flows towards the animal pole [5,41]. Thus, the animal pole of the zygote is cleared completely of yolk granules prior to the first cleavage [5,41].

### Cleaving embryo (2 hpf-7 hpf)

Diagnostic feature: Spiral cleavage pattern with individually identifiable blastomeres (scheme: Figure 1).

A detailed description of the *P. dumerilii *spiral cleavage pattern is available from Dorresteijn [5]. The first cleavage is unequal and meridional with the larger CD blastomere inheriting three times more cytoplasm than the smaller AB blastomere [5]. The second meridional cleavage is equal in the AB but unequal in the CD blastomere so that the D blastomere contains half of the initial egg volume [5]. The following cleavages are roughly equatorial in a clockwise or counterclockwise orientation and establish a canonical spiral cleavage pattern with one quartet of macromeres and four quartets of micromeres.

During cleavage, the yolk granules segregate into the macromeres and the lipid droplets at the vegetal pole fuse to form four large lipid droplets, so that at the end of cleavage each macromere contains one large lipid droplet. The formation of the four lipid droplets is a good indicator of normal development. Larvae with more or less than four lipid droplets usually develop abnormally.

### Stereoblastula - Stereogastrula (7 hpf-13 hpf)

Diagnostic feature: immobile spherical mass of dividing blastomeres whose cell lineage can no longer be followed by eye (scheme: Figure 1).

At this stage, the micromeres divide rapidly. Their overall pattern changes from spiral to bilateral symmetry and the micromeres start their epibolic movements towards the vegetal pole to envelop the macromeres. This was first observed by Wilson [42] in *Alitta succinea *(previously called *Nereis limbata*) and *Platynereis megalops *(previously called *Nereis megalops*). Dorresteijn [5], Schneider and Bowerman [43] and our own observations (A.H.L. Fischer, unpublished data) have confirmed that this is also the case for *P. dumerilii*. Through epiboly, the trochoblasts, that will give rise to the prototroch cells, come to lie in their final equatorial position. Near the vegetal pole, the mesoblasts originating from the 4 d micromere start dividing and produce the mesodermal bands.

### Protrochophore (13 hpf-24 hpf)

Diagnostic feature: pre-larva, slowly rotating in the jelly driven by the multi-ciliated equatorial prototroch cells (scheme: Figure 1).

The term "protrochophore" was introduced by Häcker [36] to refer to the earliest trochophore characterized by a broad ciliary belt. Later, this term was used more generally for very early trochophores, which slowly rotate on the substrate regardless of the belt width (e.g. [44] for *P. dumerilii *and [45] for *Chaetopterus*). Thus, the defining feature for this stage is the presence of a belt of multiciliated cells that have differentiated from the trochoblasts. The upper hemisphere apical to the ciliary belt is referred to as "episphere" and the lower hemisphere as "hyposphere". Driven by ciliary beating, the protrochophore slowly rotates inside its jelly.

The number of cells is rapidly increasing. During this stage, the stomodeal field starts to form on the prospective ventral side of the larva posterior and adjacent to the prototroch cells [5]. At the end of this stage, the stomodeal anlage is a triangular region, which contains approximately 20 cells. In the hyposphere, the mesodermal bands continue to grow. From a ventral or dorsal view, they form a v-shaped cell mass inside the larva. By the end of this stage the apical tuft - a ciliated structure at the animal pole - appears.

### Trochophore stages

Pelagic, non-feeding larvae for dispersal. Spherical shape with equatorial ciliary belt (prototroch) and apical organ. Driven by metachronic waves of beating cilia, trochophores swim in a right-handed helix while rotating around their anterior-posterior axis. Positive phototaxis. Development is highly synchronized.

### Early trochophore (24 hpf-26 hpf)

Diagnostic feature: hatching larvae actively swimming in the water column but yet without phototaxis (scheme: Figure 1).

**Figure 7:**
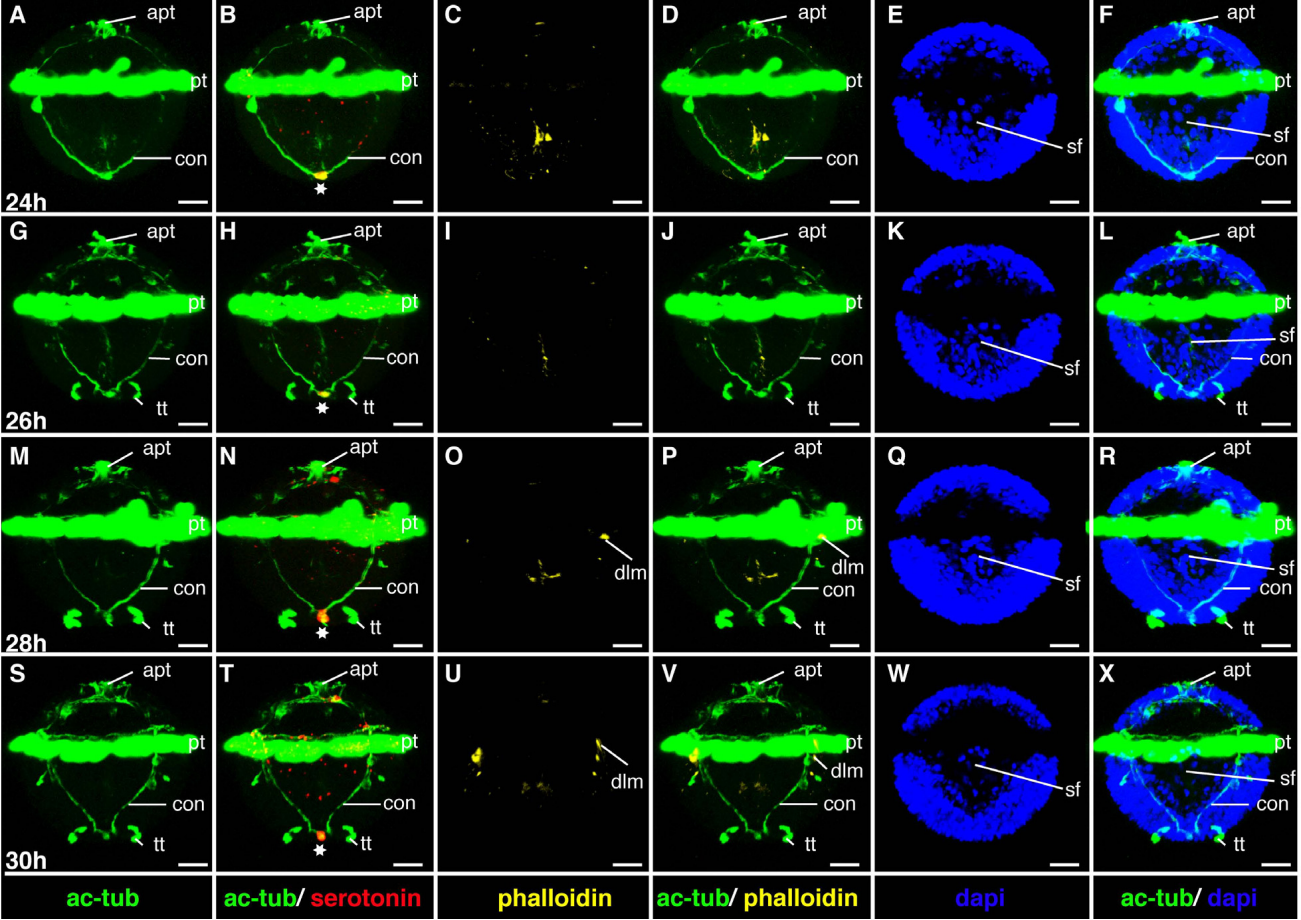
**Ventral nerve cord and muscle development of *P. dumerilii*, 24-30 hpf, ventral view, anterior up**. The age of the larvae in each row is given in the lower left corner of the first picture of each row. The displayed staining is indicated at the bottom of each column. A, G, M, S: Two axons/axon bundles, which grow from posterior to anterior, connect the posterior serotonergic cell to the prototroch ring nerve. They form the connectives (con) of the ventral nerve cord. B: The first serotonergic cell of the ventral nerve cord is located at the posterior end of the larva (white star). C, D, I, J, O, P, U, V: The dorsal longitudinal muscles are visible form 28 hpf onwards. E, K, Q, W: The stomodeal field (sf) is visible on the ventral side. F, L, R, X: The telotroch (tt) is visible at the posterior end of the larva from around 26 hpf onwards. CLSM microscopy, maximum projection, Imaris surpass mode. Scale bar in all images 20 μm. Further abbreviations see abbreviations list.

**Figure 8:**
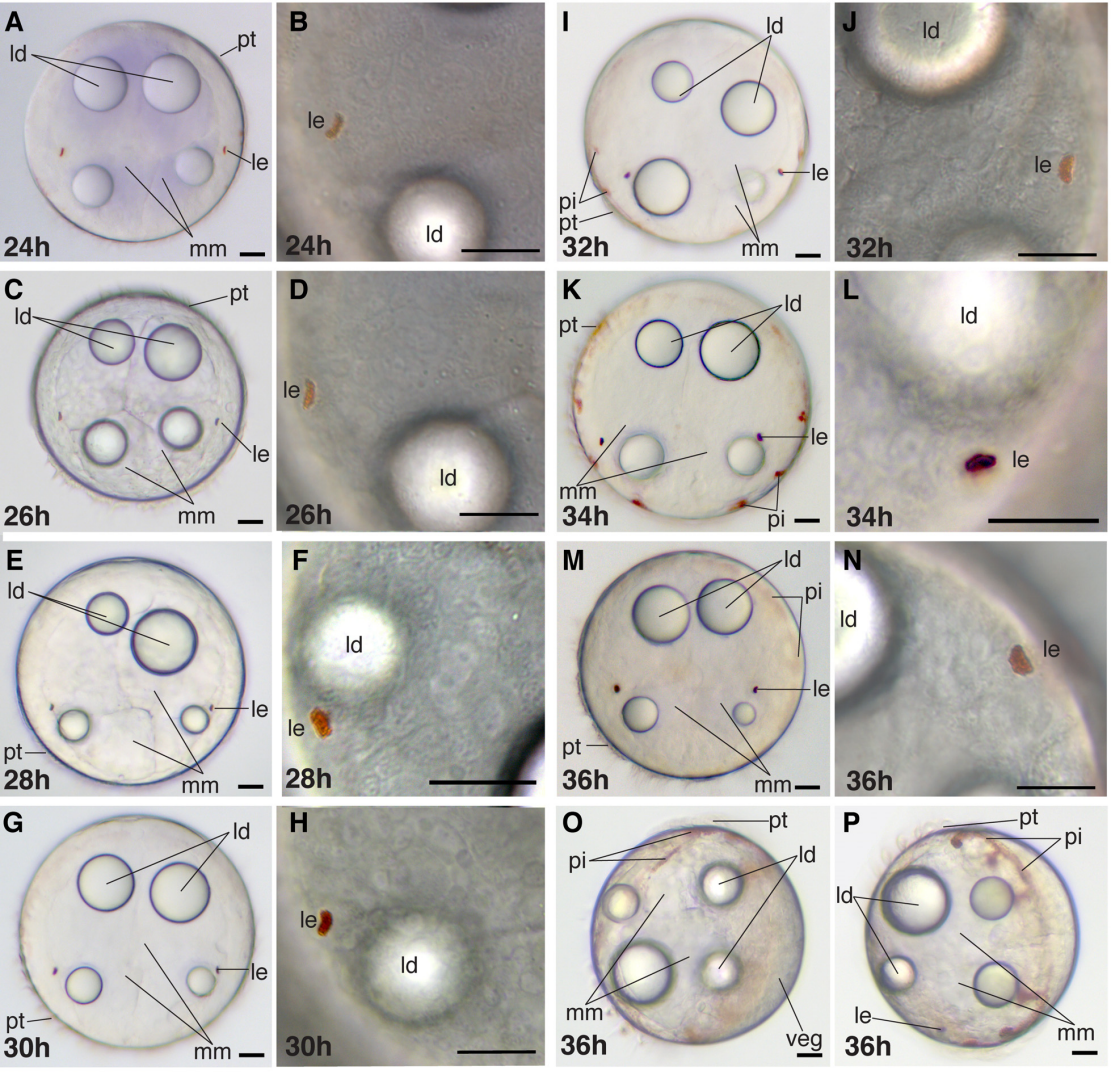
**Series of conventional light microscopy images of *P. dumerilii*, 24-36 hpf**. For each time point an overview (left) and the larval eye at higher magnification (right) is shown. All images except O and P are apical view of larvae, dorsal side up. O: Ventro-posterior view of the larva at 36 hpf, apical side left. P: Anterior-ventral view of a larva at 36 hpf, apical side left. A, C, E, G, I, K, M, O: In all larva the 4 macromeres (mm) in the center of the larva is visible. Each macromere contains one lipid droplet (ld). The two ventral lipid droplets are smaller than the dorsal ones. Around the prototroch additional pigment (pi) is visible in some larvae (I, K, M, O). B, D, F, H, J, L, N, P: The pigmentation of the larval eyes (le) becomes more intense over time. Scale bar in all images 20 μm. Further abbreviations see abbreviations list. In this and all other figure plates, fresh samples were used at every time point. Each specimen was collected from 18°C ± 0.1°C source immediately before imaging.

At this stage, the larvae "hatch" from the jelly envelope as the jelly degrades and the early trochophore larvae start swimming. The shape of an early trochophore remains spherical; episphere and hyposphere have similar size (Figure 7[E, F]). Pigment first appears in the shading pigment cell of the larval eyes but the amount of pigment remains minute and difficult to detect (Figure 8[A, B]). The larval eyes are located laterally on the episphere (Figure 8[A, B]). The anlage of the stomodeum - the stomodeal field - retains its triangular shape on the ventral side in the hyposphere (Figure 7[E, F]).

At approximately 25 hpf, an additional band of ciliated cells, the telotroch, differentiates at the posterior end of the larva, while leaving a gap on the dorsal and ventral side (Figure 7[G, L]). The telotroch appears especially pronounced in anti-acetylated tubulin stainings but can also be observed in living specimens with conventional light microscopy (data not shown).

The telotroch marks the anterior border of the pygidium - the posterior end of the larvae - and separates it from the rest of the trunk.

#### Characteristic features in the nervous system and musculature

**Figure 9:**
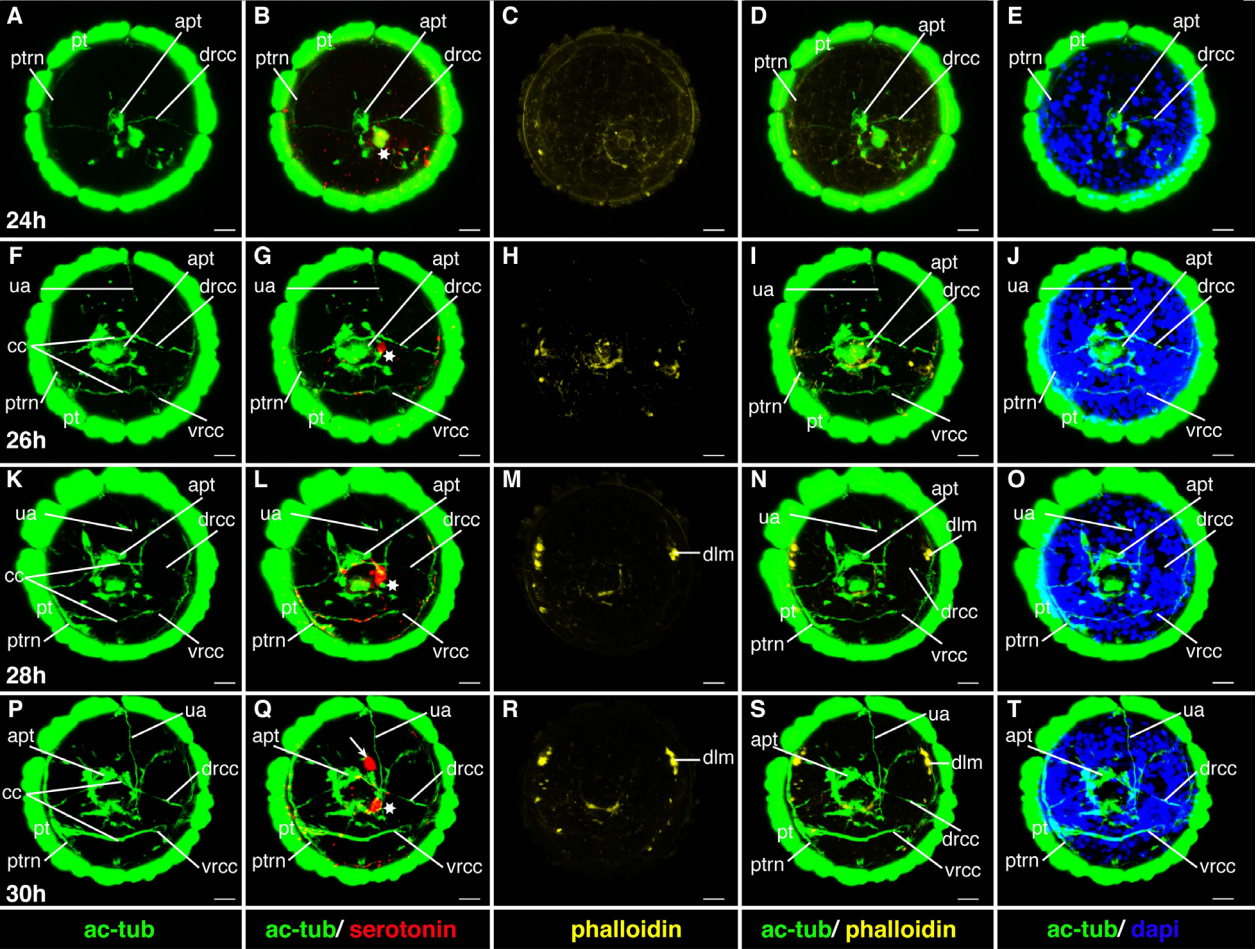
**Brain and muscle development of *P. dumerilii*, 24-30 hpf, apical view, dorsal side up**. The age of the larvae in each row is given in the lower left corner of the first picture of each row. The displayed staining is indicated at the bottom of each column. A, F, K, P: The dorsal root of the circumesophageal connectives (drcc) is visible at 24 hpf as well as the developing prototroch ring nerve (ptrn). From 26 hpf onwards the ventral branch of the circumesophageal connectives (vrcc) and the unpaired dorsal axon (ua) are visible. The dorsal and ventral roots of the circumesophageal connectives connect the cerebral commissures (cc) in the brain. The prototroch ring nerve becomes more pronounced. B, G, L: The first serotonergic cell (white star) is located in the apical organ just below the apical tuft (apt). Q: At 30 hpf a second serotonergic cell appears (white arrow). C, D, H, I, M, N, R, S: From 28 hpf onwards the dorsal longitudinal muscles (dlm) become visible. E, J, O, T: The amount of cells in the brain is increasing. CLSM microscopy, maximum projection, Imaris surpass mode. Scale bar in all images 20 μm. Further abbreviations see abbreviations list.

The apical ganglion, the larval brain, forms beneath the apical tuft (Figure 9[A, B]). The apical ganglion contains only one serotonergic cell (detected by the 5-HT antibody) and a few other neurons (detectable by an antibody directed against acetylated alpha-tubulin), of which two send axons to the prototroch nerve (Figure 9[B]). These axons are the first axons of the larval brain. The prototroch ring nerve develops at this stage and runs beneath the prototroch cells (Figure 9[A]). The ventral nerve cord has developed a v-shaped pair of axons, which grow from posterior to anterior and connect a single serotonergic cell located at the posterior end of the larva to the prototroch nerve (Figure 7[A, B]). No differentiated muscles are yet detectable by phalloidin staining (Figure 7[C, D] and Figure 9[C, D]).

### Mid-trochophore 26 hpf-40 hpf

Diagnostic feature: free-swimming trochophores showing phototaxis (scheme: Figure 2).

The beginning of this stage is marked by the onset of phototaxis. Phototactic steering and the neurobiology underlying *P. dumerilii *swimming and phototaxis have been characterized in mechanistic and molecular detail by Jékely *et al. *[2] and can be observed until around the mid-nectochaete stage.

**Figure 10:**
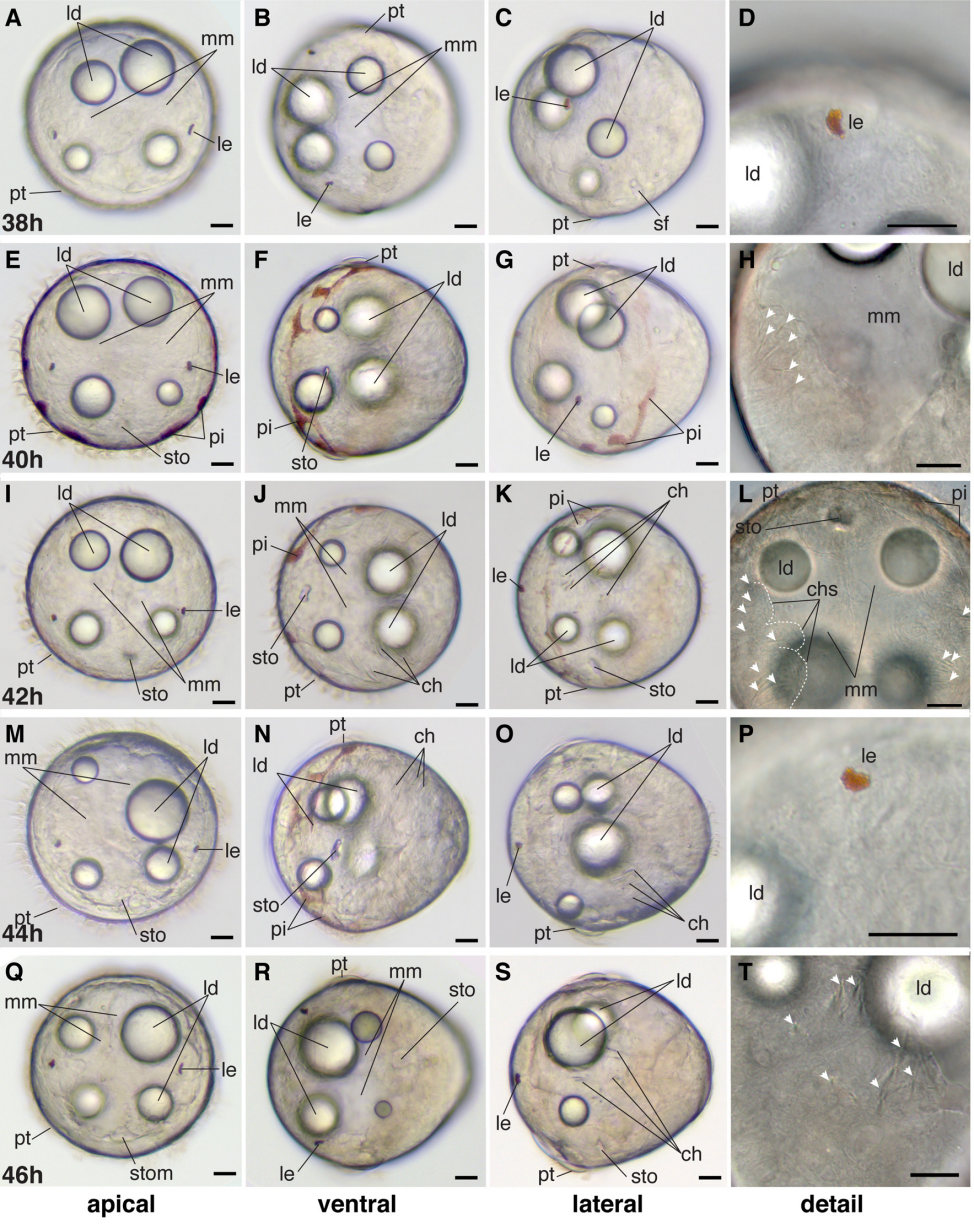
**Series of conventional light microscopy images of *P. dumerilii*, 38-46 hpf**. The age of the larvae in each row is given in the lower left corner of the first picture of each row. To give an overview on the morphological changes throughout development, for each time point an apical view of larvae, dorsal side up (first column: A, E, I, M, Q), ventral view, anterior side left (second column: B, F, J, N, R), lateral view, anterior side left and dorsal side up (third column: C, G, K, O, S) and several details (fourth column: D, H, L, P, T) are shown. H: From 40 hpf onwards the chaetae (ch) (white arrow heads) can be seen inside the trunk. L, T: The chaetae (white arrow heads) grow out from the chaetal sacs and increase in length (chs). L: The chaetal sacs are marked with a white stippled line on the right side of the larva. E, N: The stomodeal opening is visible. Note that the intensity of the pigmentation pattern may vary substantially around the prototroch and posterior end among trochophore larvae. F, J, N: Individuals with a high amount of pigment, whereas the individuals in B and R show almost no pigment. Scale bar in all images 20 μm. Further abbreviations see abbreviations list.

At the mid-trochophore stage, the larval eyes become more prominent due to an increased amount of shading pigment and they develop the characteristic cup shape (Figure 8[D, F, H, J, L, N], and Figure 10[D]) [46]. In addition to the red pigment of the larval eyes, red pigment spots around the prototroch cells often appear. Appearance and amount of pigment varies between batches and even between individuals (Figure 8[C, E, G, I, K, M, O, P], and Figure 10[A-C, E-G]).

**Figure 11:**
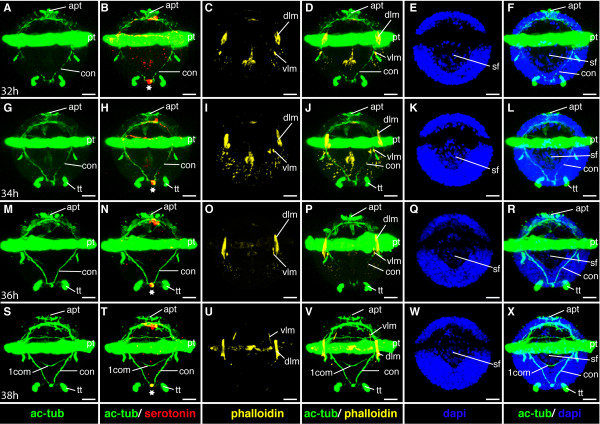
**Ventral nerve cord and muscle development of *P. dumerilii*, 32-38 hpf, ventral view, anterior up**. The age of the larvae in each row is given in the lower left corner of the first picture of each row. The displayed staining is indicated at the bottom of each column. A, G, M, S: The connectives (con) show an increasing staining intensity (compare the tubulin staining at the different time points). S: The first commissure (1com) of the ventral nerve cord is formed. B, H, N, T: Only one serotonergic cell is visible in the ventral nerve cord (white star). C, D, G, D, I, J, O, P, U, V: The dorsal longitudinal muscles (dlm) increase in length and the ventral longitudinal muscles (vlm) become clearly visible by phalloidin staining at 32 hpf. E, F, K, L, Q, R, W, X: The stomodeal field (sf) gets located more anteriorly between 32 hpf and 38 hpf and cells start to form a circle in the stomodeal field. CLSM microscopy, maximum projection, Imaris surpass mode. Scale bar in all images 20 μm. Further abbreviations see abbreviations list.

The number of cells in the stomodeal field slowly increases. These cells start to form a ring around the stomodeal opening - the stomodeal rosette (Figure 7[K, Q, W], and Figure 11[E, K, Q, W]). The stomodeal rosette moves anteriorly so that it comes to lie just posterior the prototroch by the end of this stage (Figure 11[W]). During this stage, overall larval morphology changes gradually from spherical to conical, thus initiating the elongation of the trunk (Figure 7[K, Q, W], and Figure 11[E, K, Q, W]). This process continues and becomes more apparent throughout subsequent larval development.

#### Characteristic features in the nervous system and musculature

In the mid-trochophore, the first cerebral commissure forms, interconnecting the ventral branches of the paired circumesophageal connectives (Figure 9[F, G, K, L]). The circumesophageal connectives are the anterior part of the connectives - they form the connection between the brain and the ventral nerve cord. The division of the circumesophageal connectives into a dorsal and ventral root is a typical feature of the annelid brain [47], which is also present in *P. dumerilii*.

**Figure 12:**
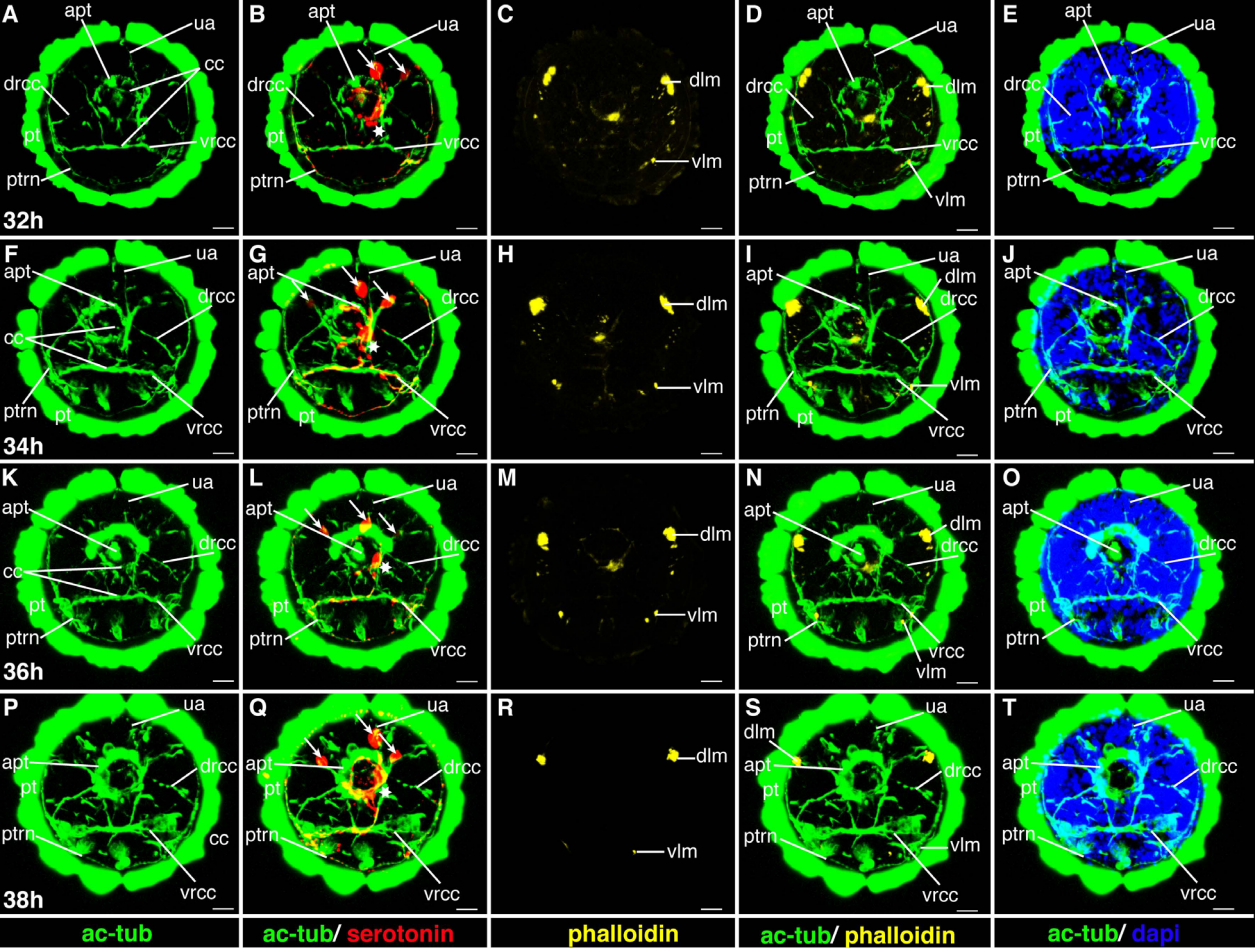
**Brain and muscle development of *P. dumerilii*, 32-38 hpf, apical view, dorsal side up**. The age of the larvae in each row is given in the lower left corner of the first picture of each row. The displayed staining is indicated at the bottom of each column. A, F, K, P: The dorsal and ventral branch of the circumesophageal connectives (drcc and vrcc) become thicker and the prototroch ring nerve (ptrn) is well visible. The dorsal and ventral roots of the circumesophageal connectives connect the cerebral commissures (cc). B, G, L, Q: Additionally to the first and second serotonergic cell (white star and white arrow respectively) two more serotonergic cells develop laterally in the brain of the larvae (white arrows). C, D, H, I, M, N, R, S: The dorsal longitudinal muscles (dlm) become more pronounced. The ventral longitudinal muscles (vlm) become clearly visible around 32 hpf. E, J, O, T: The amount of cells on the ventral side of the episphere is increasing. CLSM microscopy, maximum projection, Imaris surpass mode. Scale bar in all images 20 μm. Further abbreviations see abbreviations list.

A single asymmetric unpaired axon (ua) develops on the dorsal side of the larval episphere (Figure 9[K, P]). Three more serotonergic cells can be detected around the apical ganglion (Figure 9[Q], and Figure 12[B, G, L, Q]). At the end of the mid-trochophore corresponding to approximately 38 hpf the first commissure forms in the ventral nerve cord (Figure 11[S, T]), ([8] and our own observation).

At the beginning of this stage at around 28 hpf the dorsal longitudinal muscles appear, while the ventral longitudinal muscles become clearly visible slightly later, at around 32 hpf (Figure 7[O, P, U, V], Figure 9[M, N, R, S], Figure 11[C, D], and Figure 12[C, D]).

### Late trochophore 40 hpf-48 hpf

Diagnostic features: Distinct stomodeal opening surrounded by stomodeal rosette, first chaetae visible inside the trunk as first sign of larval segmentation (scheme: Figure 2).

The stomodeal rosette moves further towards the anterior until it extends partially into the episphere and the stomodeal opening - the mouth - becomes visible by light microscopy just posterior to the prototroch (Figure 10[F]).

Three larval segments appear simultaneously. They are easily identified by the developing chaetae in the trunk (Figure 10[E]). The chaetae in the first and second segment are slightly advanced in development compared to the chaetae in the third segment. The chaetae start growing from pouches, the chaetal sacs, which are positioned laterally on both sides deep inside the trunk (Figure 10[H, J-L, N, O]). Therefore, the chaetae first appear inside of the animal, where they grow rapidly in length. Each of the three chaetigerous segments develops a ventral and a dorsal set of chaetal sacs producing chaetae (Figure 10[K, S]). The trunk further elongates (Figure 10[F, G, J, K, N, O, R, S]), so that the macromeres change their shape and become narrower towards the posterior. The larval eyes remain the only pair of eyes until the end of this stage (e.g. Figure 10[D, P]).

Pigmentation surrounding the prototroch can be greater than in the previous stage, but is still highly variable (Figure 10[E-G, I-K, M-O, Q-S]).

#### Characteristic features in the nervous system and musculature

**Figure 13:**
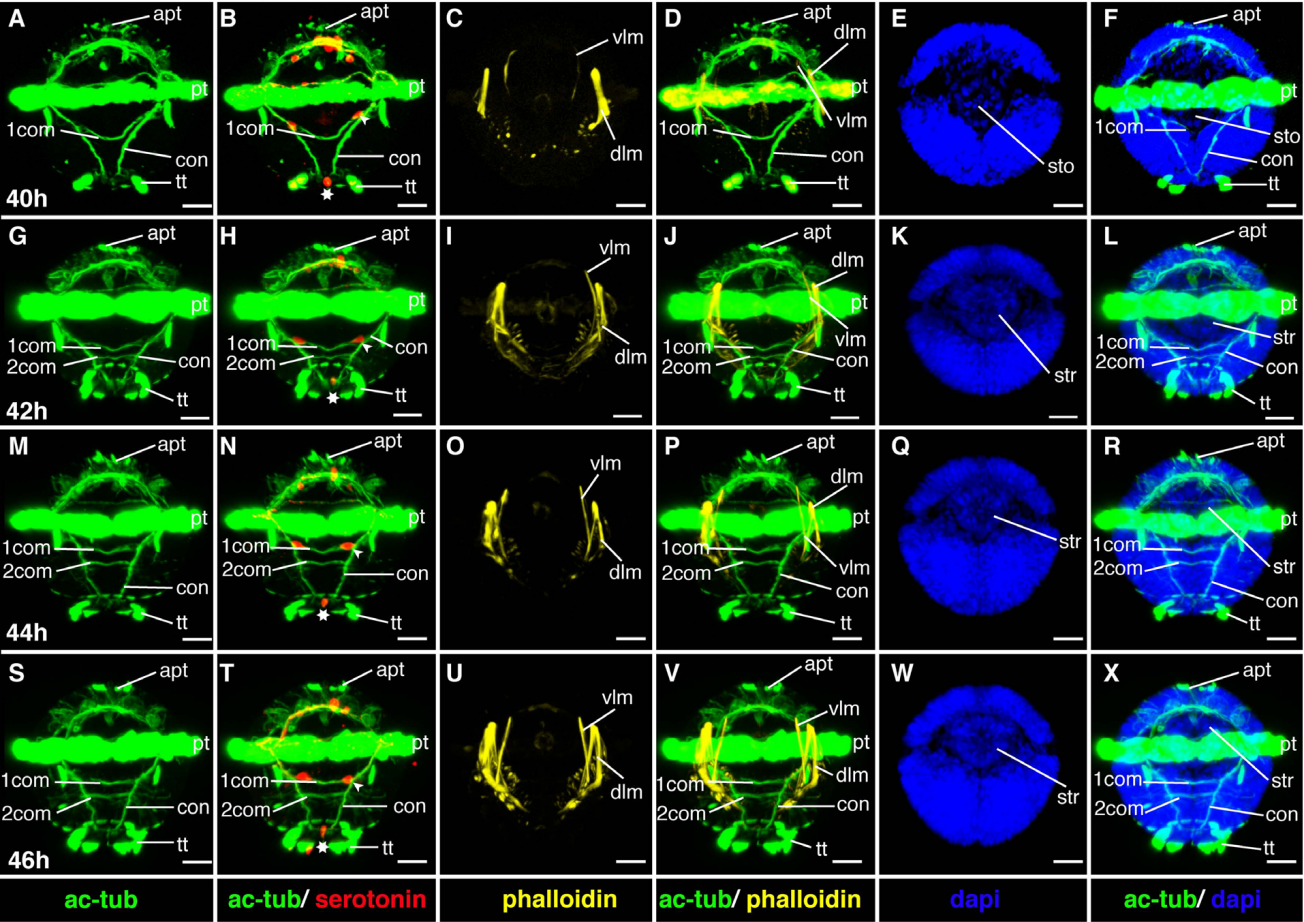
**Ventral nerve cord and muscle development of *P. dumerilii*, 40-46 hpf, ventral view, anterior up**. The age of the larvae in each row is given in the lower left corner of the first picture of each row. The displayed staining is indicated at the bottom of each column. A, G, M, S: The second commissure (2com) of the ventral nerve cord is formed at 42 hpf. B, H, N, T: In addition to the unpaired serotonergic cell at the posterior end of the larva (white star), a pair of serotonergic neurons becomes visible at the first commissure (white arrow head). C, D, I, J, O, P, U, V: The dorsal and ventral longitudinal muscles (dlm and vlm) increase rapidly in length. E, F, K, L, Q, R, W, X: The stomodeal rosette (str) is formed and gets into a more anterior position. CLSM microscopy, maximum projection, Imaris surpass mode. Scale bar in all images 20 μm. Further abbreviations see abbreviations list.

**Figure 14:**
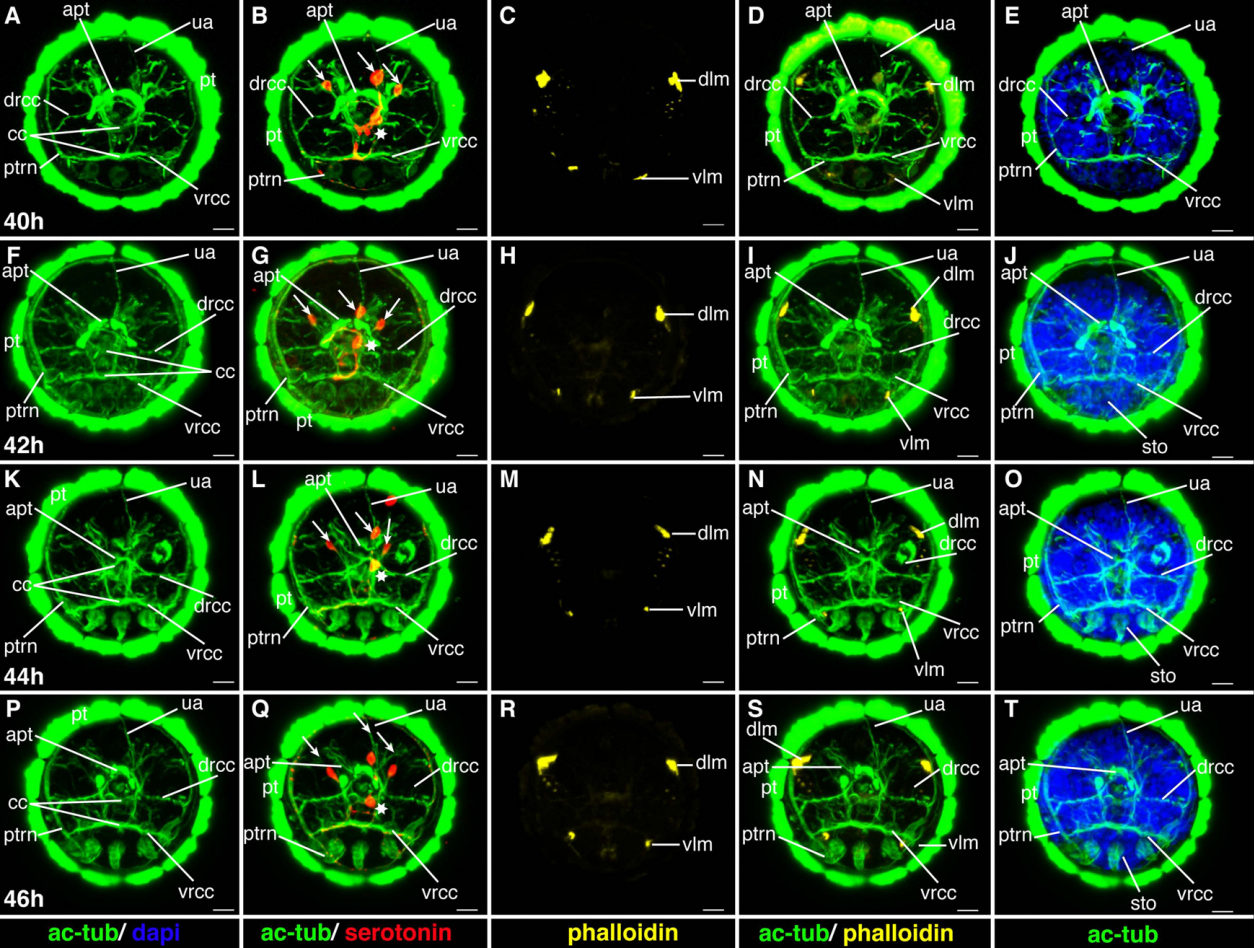
**Brain and muscle development of *P. dumerilii*, 40-46 hpf, apical view, dorsal side up**. The age of the larvae in each row is given in the lower left corner of the first picture of each row. The displayed staining is indicated at the bottom of each column. A, F, K, P: The dorsal and ventral branch of the circumesophageal connectives (drcc and vrcc), the cerebral commissures (cc) and the dorsal unpaired axon (ua) are well visible, the prototroch ring nerve (ptrn) is well developed. B, G, L, Q: Four serotonergic cells are visible in the brain (the central one marked with a white star, the more dorsal ones marked with white arrows). G, D, H, I, M, N, R, S: The dorsal longitudinal muscles (dlm) and ventral longitudinal muscles (vlm) are intensely stained. E, J, O, T: The stomodeum (sto) becomes visible in the episphere from around 42 hpf onwards. CLSM microscopy, maximum projection, Imaris surpass mode. Scale bar in all images 20 μm. Further abbreviations see abbreviations list.

Towards the end of this stage at 44 hpf, the second commissure forms in the ventral nerve cord (Figure 13[G, H]). The dorsal and ventral longitudinal muscles increase in length and thickness (Figure 13[C, D, I, J, O, P, U, V]). The dorsal longitudinal muscles develop a second more medial branch. At the end of this stage, the first signs of oblique and parapodial muscles become visible. The brain appears more complex due to an increasing number of neurites (Figure 14[A, B, D-G, I-K, L, N-Q, S, T]).

### Metatrochophore stages

Conical three-segmented larva, non-feeding with solely pelagic lifestyle. Retains the lifestyle of the trochophore with helical swimming and positive phototaxis, while bilateral, segmental structures such as chaetae, ciliary bands, commissures and various muscles rapidly develop. This reflects the transitory nature of the metatrochophore stages - transforming the trochophore into a nectochaete larva. Development is highly synchronized.

### Early metatrochophore (48 hpf-51 hpf)

Diagnostic feature: formation of the first paratroch, chaetae reach the body wall (scheme: Figure 2).

**Figure 15:**
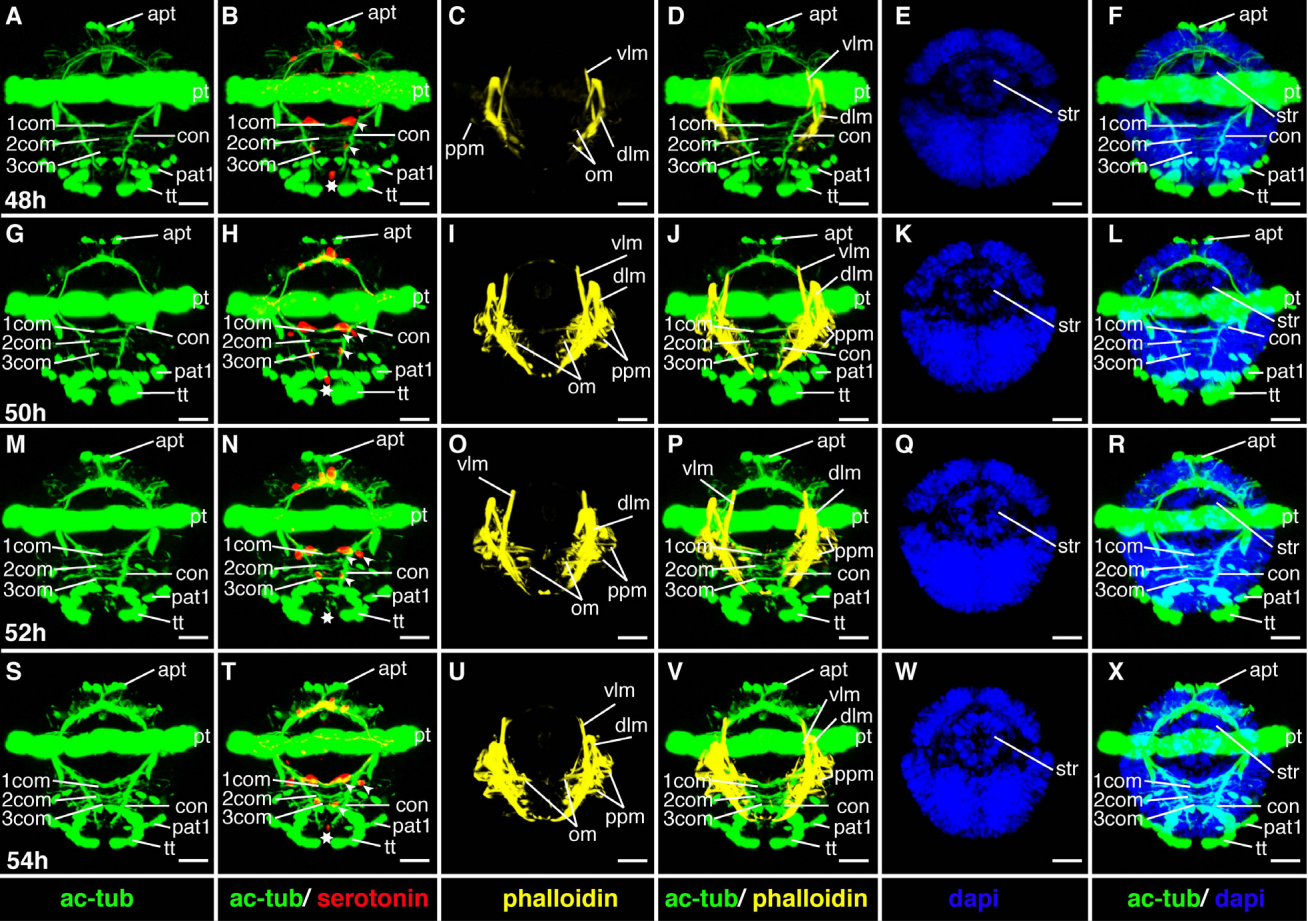
**Ventral nerve cord and muscle development of *P. dumerilii*, 48-54 hpf, ventral view, anterior up**. The age of the larvae in each row is given in the lower left corner of the first picture of each row. The displayed staining is indicated at the bottom of each column. A, G, M, S: The third commissure (3com) of the ventral nerve cord is formed at 48 hpf and more commissural axons are visible in the ventral nerve cord (e.g. compare A, G, M and S). B, H, N, T: In addition to the unpaired serotonergic cell at the posterior end of the larva (white star) and the first pair of serotonergic neurons at the first commissure. A pair of serotonergic cells near the second commissure and a second pair at the first commissure become visible (white arrow heads). E, K, Q, W: The stomodeal rosette (str) is well visible and contains more cells over time. C, D, I, J, O, P, U, V: Parapodial muscles (ppm) and oblique muscles (om) become from 48 hpf onwards. F, L, R, X: The first paratroch (pat1) is visible at the posterior boarder of the second chaetigerous segment from around 48 hpf onwards. CLSM microscopy, maximum projection, Imaris surpass mode. Scale bar in all images 20 μm. Further abbreviations see abbreviations list.

The early metatrochophore stage starts with the formation of a paratroch at the posterior border of the second chaetigerous segment (Figure 15[F]). Paratrochs are ciliary bands posterior to the prototroch [48]. They are typical for nereidid larvae and develop at the posterior boarder of the first and second chaetigerous segments, e.g. [49,50]. The first paratroch does not surround the whole body, but leaves a gap on the dorsal and ventral sides (Figure 15[F, L]).

The chaetigerous trunk segments are well-defined at this stage: the chaetae are well developed and are poised to break through the body wall (Figure 10[B, F]). Commissures, oblique muscles and parapodial muscles are forming in each segment (see below). Thus, this stage represents the beginning of the metatrochophore larva, following Gravely's definition [48].

The trunk continues to elongate and the conical shape of the macromeres becomes even more apparent. The number of cells contributing to the stomodeal rosette slowly increases throughout subsequent larval development. The rosette is positioned medially in the prototroch region on the ventral side (Figure 10[B, F], and Figure 15[E, K]).

In addition to the red pigment of the eyes and surrounding the prototroch, the region of the telotroch can exhibit some red pigment (Figure 10[B, F]). As is the case for the pigmentation around the prototroch, the amount and appearance of pigment varies between individuals.

#### Characteristic features in the nervous system and musculature

**Figure 16:**
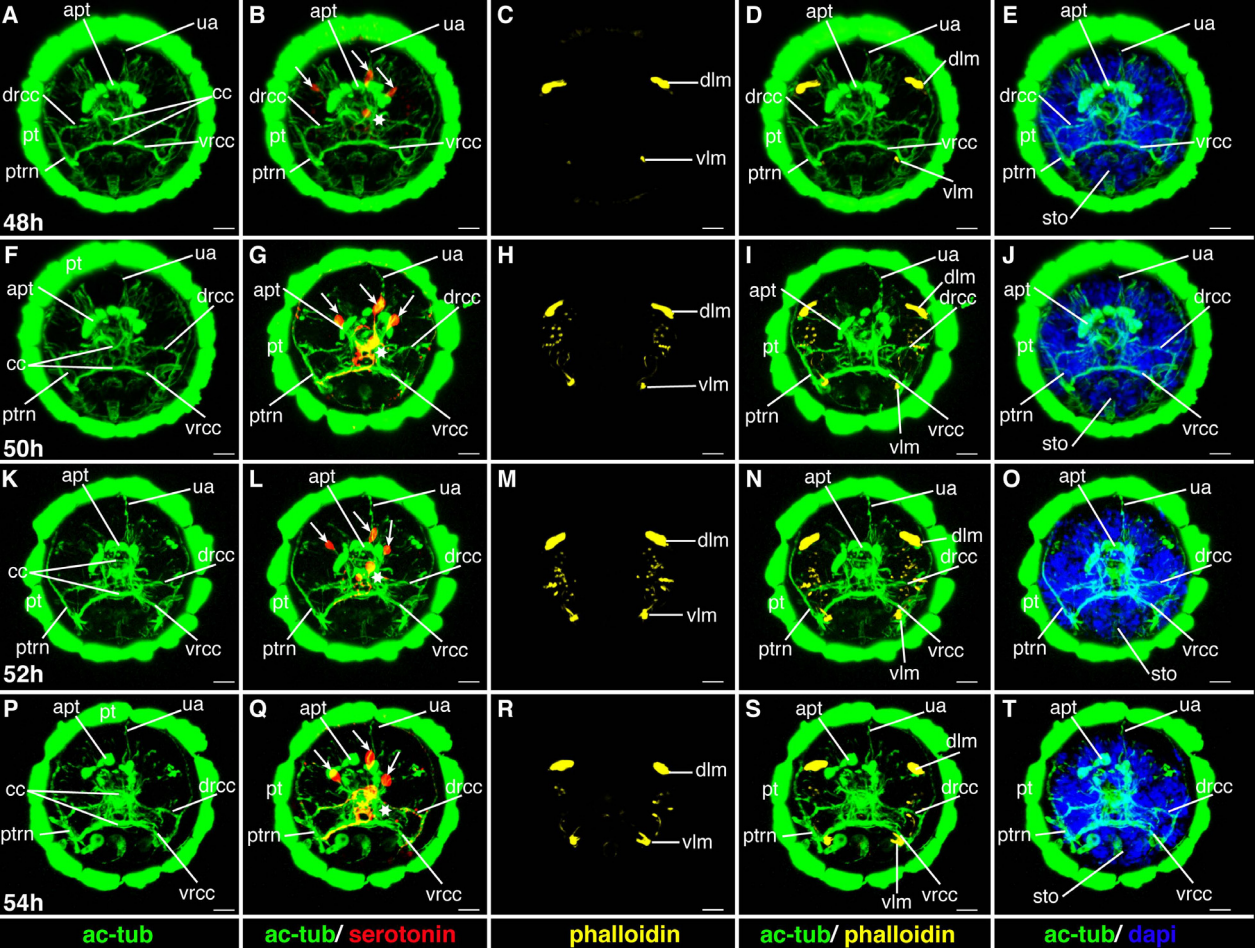
**Brain and muscle development of *P. dumerilii*, 48-54 hpf, apical view, dorsal side up**. The age of the larvae in each row is given in the lower left corner of the first picture of each row. The displayed staining is indicated at the bottom of each column. A, F, K, P: The dorsal and ventral branches of the circumesophageal connectives (drcc and vrcc) approach each other. The distance between the prototroch ring nerve (ptrn) and the prototroch (pt) begins to increase and the prototroch ring nerve approaches the cerebral commissures (cc) (e.g. compare A, F, K and P). B, G, L, Q: Four serotonergic cells are visible in the brain (the central one marked with a white star, the more dorsal ones marked with white arrows). C, D, H, I, M, N, R, S: The dorsal longitudinal muscles (dlm) and ventral longitudinal muscles (vlm) are intensely stained. E, J, O, T: The stomodeum is well visible in the episphere. CLSM microscopy, maximum projection, Imaris surpass mode. Scale bar in all images 20 μm. Further abbreviations see abbreviations list.

The third commissure in the ventral nerve cord forms (Figure 15[A, G]). The brain grows in size and appears more complex (Figure 16[A, F]). A second and third pair of serotonergic cells develops in the ventral nerve cord (Figure 15[B, H]). The oblique muscles elongate and become visible, as is also the case for the parapodial muscles (Figure 15[C, I]). This process continues from this stage onwards.

### Mid-metatrochophore (51 hpf-60 hpf)

Diagnostic feature: pigment of the adult eyes clearly visible, chaetae outside the body wall but parapodia not yet formed (scheme: Figure 2).

**Figure 17:**
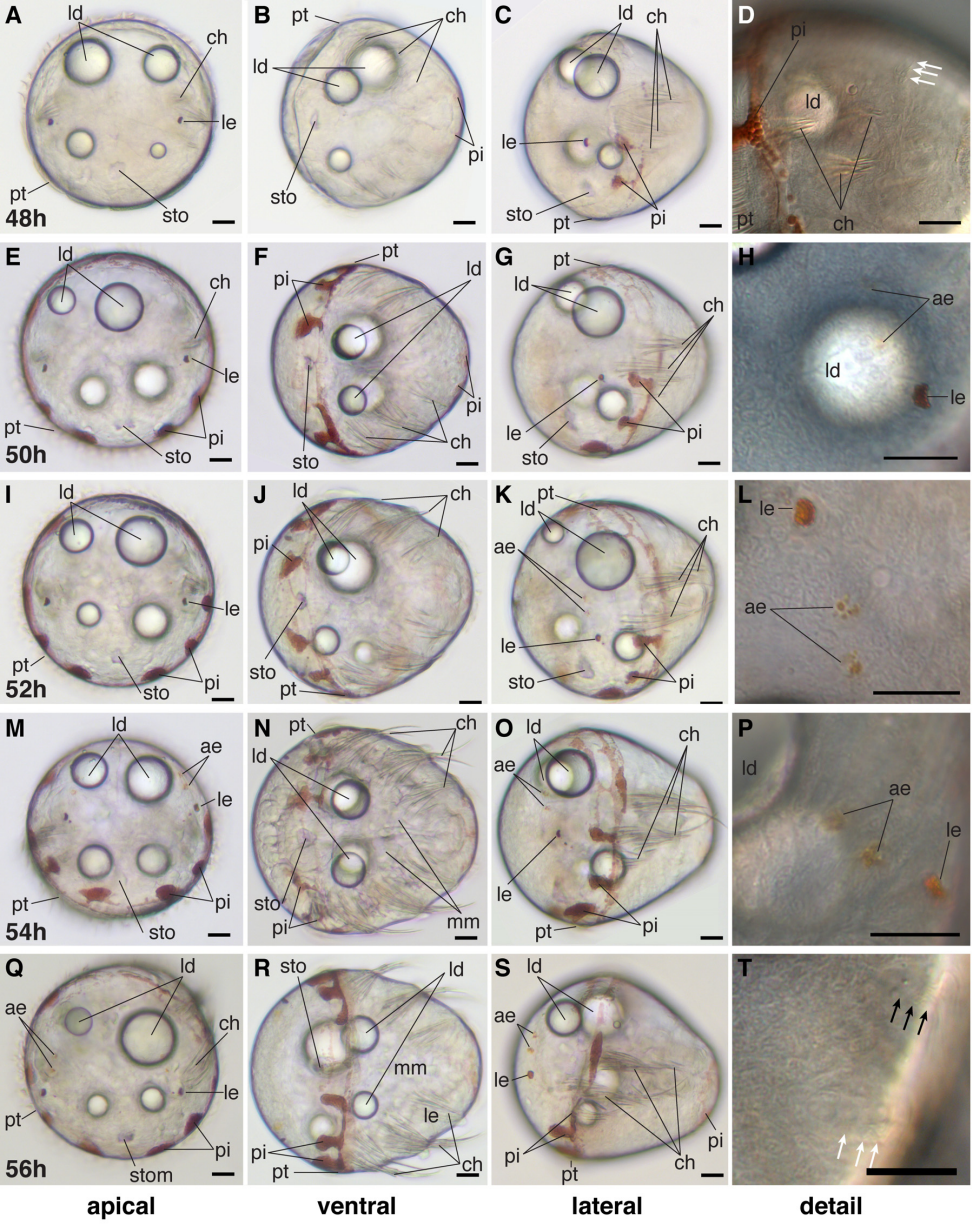
**Series of conventional light microscopy images of *P. dumerilii*, 48-56 hpf**. The arrangement of the images is similar to figure 10. H: At 50 hpf the adult eye pigment (ae) appears very faintly. L, M, O, P, Q, S: Adult eye pigment becomes well visible from 52 hpf onwards. J: The chaetae (ch) break through the body wall at 52 hpf. F, J, N, R: Chaetae increase rapidly in length. T: Additionally to the first paratroch (with arrows) the second paratroch (black arrows) develop at the posterior boarder of the first segment. Scale bar in all images 20 μm. Further abbreviations see abbreviations list.

**Figure 18:**
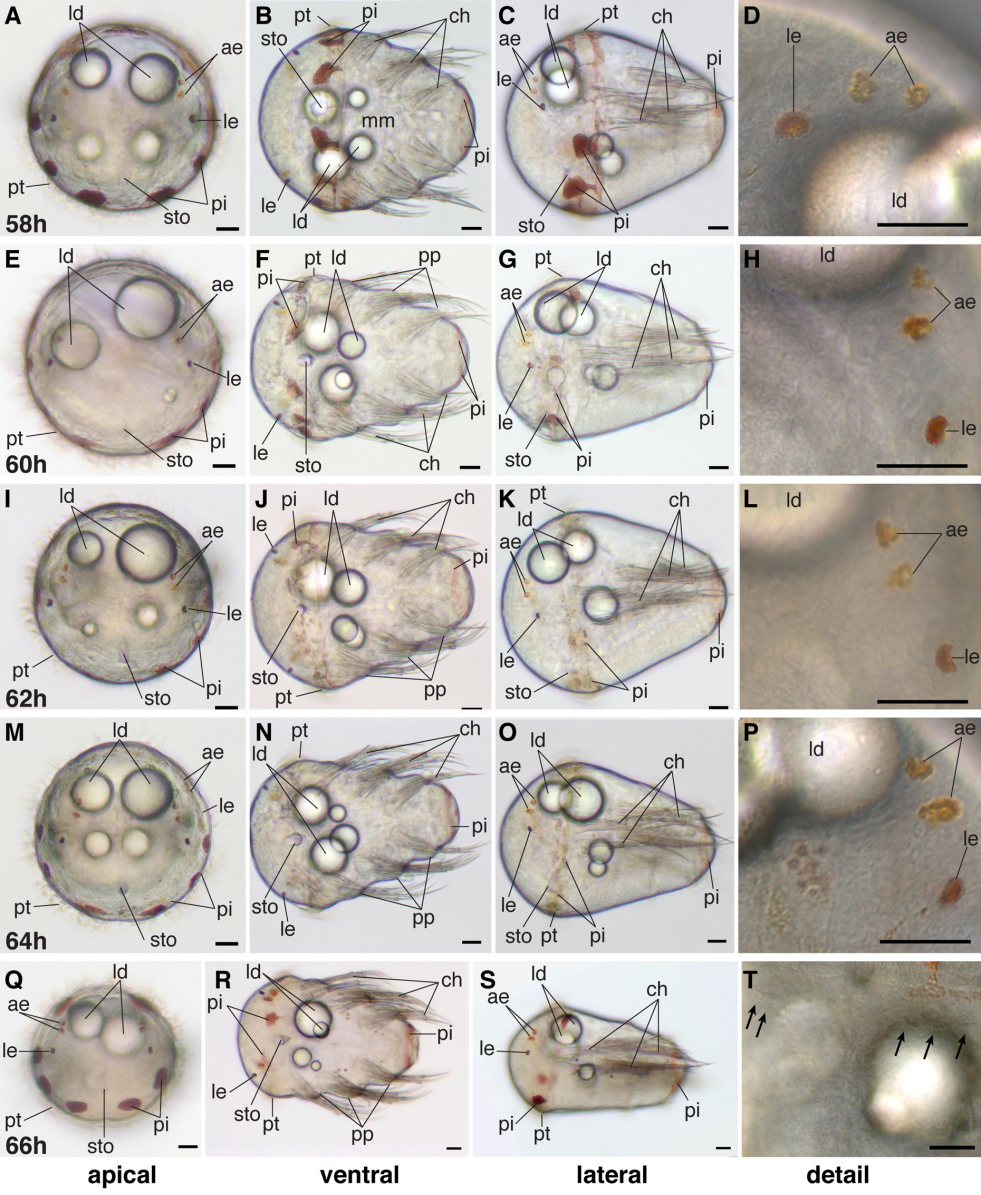
**Series of conventional light microscopy images of *P. dumerilii*, 58-66 hpf**. The arrangement of the images is similar to figure 10. B, F, J, N, R: The chaetae (ch) rapidly increase in length and the chaetae of the third segment reach the posterior end of the larva, which marks the beginning of the late metatrochophore stage. F, J, N, R: Thee pairs of parapodia (pp) start forming laterally at the trunk. D, H, L, P: The amount of pigment in the adult eyes (ae) increases. T: The metatroch (black arrows) forms posterior to the prototroch at 66 hpf. Scale bar in all images 20 μm. Further abbreviations see abbreviations list.

The mid-metatrochophore stage is marked by the appearance of two additional pairs of eyes - the adult eyes, located lateral-dorsally in the episphere (Figure 17[L, M, O-Q, S], and Figure 18[A, C, D]). They are barely detectable in the beginning due to their small amount of pigment. However, during this stage, the amount of pigment in the adult eyes increases substantially, so that they are easily identifiable (Figure 17[L, M, O-Q, S], and Figure 18[A, C, D]). One pair of adult eyes is located more dorsally, the other one more ventrally (Figure 17[L, M, O-Q, S], and Figure 18[A, C, D]).

The beginning of this stage is also marked by the chaetae that break through the body wall (Figure 17[J]). At the positions where they penetrate, slight elevations of the ectodermal cell layer are the first indications of parapodia formation. During this stage, the chaetae increase significantly in length (Figure 17[N, R], and Figure 18[B]). Only the chaetae in the third chaetigerous segment grow out more slowly and remain shorter than those in the more anterior segments (Figure 17[J, N, R], and Figure 18[B]).

**Figure 19:**
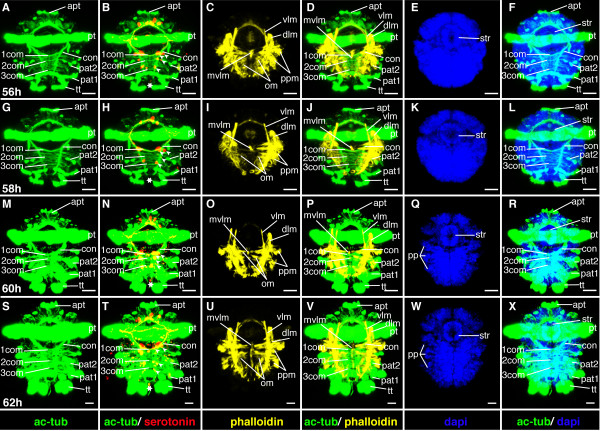
**Ventral nerve cord and muscle development of *P. dumerilii*, 56-62 hpf, ventral view, anterior up**. The age of the larvae in each row is given in the lower left corner of the first picture of each row. The displayed staining is indicated at the bottom of each column. A, G, M, S: An increasing amount of commissural axons becomes visible along the ventral nerve cord (compare A, G, M, S). B, H, N, T: A pair of serotonergic cells becomes visible in the third segment (white arrow heads), additionally to the unpaired serotonergic cell (white star) and other serotonergic cells in the first and second segment (white arrow heads). C, D, I, J, O, P, U, V: Parapodial muscles (ppm) and oblique muscles (om) increase rapidly in size. The unpaired medial ventral longitudinal muscle (mvlm) becomes visible posterior to the stomodeum and elongates posteriorly (compare C, I, O, U). E, K, Q, W: The stomodeal rosette (str) is well visible. Three pairs of parapodia (pp) become visible from around 60 hpf onwards. F, L, R, X: The second paratroch (pat2) becomes visible at the posterior boarder of the first chaetigerous segment from 56 hpf onwards. CLSM microscopy, maximum projection, Imaris surpass mode. Scale bar in all images 20 μm. Further abbreviations see abbreviations list.

As the trunk starts to elongate and the gut anlage becomes visible, the macromeres adopt a pointed shape toward the posterior end (Figure 17[J, N, R], and Figure 18[B]). An additional ring of distinct cells forms around the stomodeal rosette (Figure 15[Q, W], and Figure 19[E, K]). The lumen of the stomodeum begins to invaginate into the head.

A second paratroch forms anterior to the first, at the posterior border of the first chaetigerous trunk segments (Figure 19[F, L]).

#### Characteristic features in the nervous system and musculature

The number of axons in the commissures increases remarkably, so that the commissures appear much broader (Figure 15[M, N, S, T], and Figure 19[A, B, G, H]). Many axons project from the ventral nerve cord into the lateral parts of the ventral plate. During this stage, convergent extension movements take place in the ventral neuroectoderm [23]. Medio-lateral intercalations of neuroectodermal cells relocate lateral cells to a more medial position within the ventral plate. Concomitantly, axonal projections from the ventral nerve cord are redirected ventrally towards the surface [23]. This process continues until 72 hpf [23].

**Figure 20:**
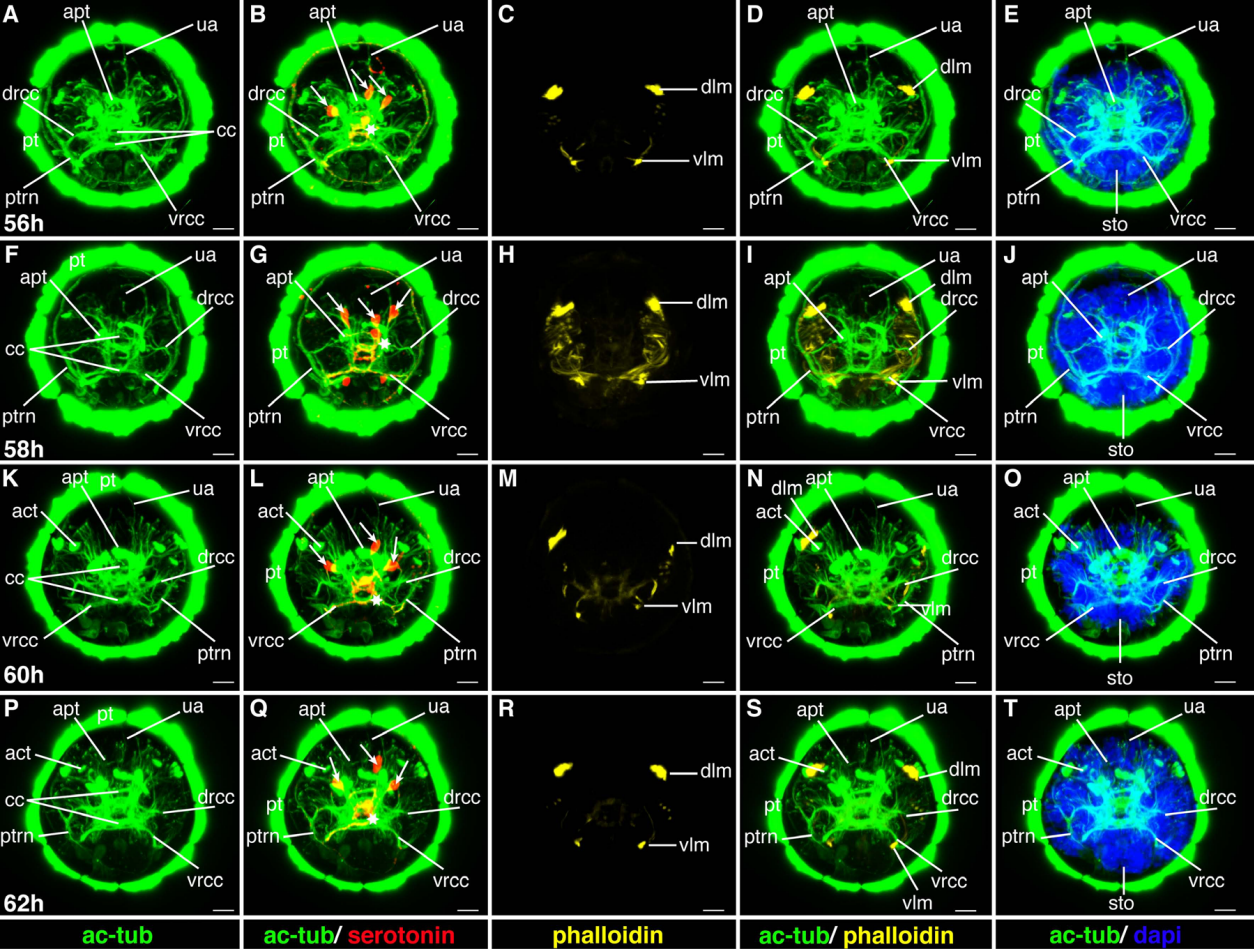
**Brain and muscle development of *P. dumerilii*, 56-62 hpf, apical view, dorsal side up**. The age of the larvae in each row is given in the lower left corner of the first picture of each row. The displayed staining is indicated at the bottom of each column. A, F, K, P: The dorsal and ventral branches of the circumesophageal connectives (drcc and vrcc) continue to approach each other. The distance between the prototroch ring nerve (ptrn) and the prototroch (pt) increases (compare A, F, K, P). B, G, L, Q: Four serotonergic cells are visible in the brain (the central one marked with a white star, the more dorsal ones marked with white arrows). C, D, H, I, M, N, R, S: The dorsal longitudinal muscles (dlm) and ventral longitudinal muscles (vlm) are intensely stained. E, J, O, T: Dorsally, the first ciliated tufts of the akrotroch (act) develop (compare J and O). The akrotroch is a row of ciliated cells, which goes laterally from the prototroch dorso-medially onto the head. CLSM microscopy, maximum projection, Imaris surpass mode. Scale bar in all images 20 μm. Further abbreviations see abbreviations list.

A fourth pair of serotonergic cells develops in the ventral nerve cord (Figure 19[B, H]). The dorsal and ventral roots of the circumesophageal connectives approach each other and the brain becomes more compact, while the prototroch nerve approaches the brain (Figure 16[K, L, N, P, Q, S], and Figure 20[A, B, D, F, G, I]). This process continues throughout the following stages. An additional longitudinal muscle, the ventral medial longitudinal muscle, begins to form along the ventral midline, starting in the first chaetigerous segment and elongating toward the posterior (Figure 19[C, D, I, J]).

### Late metatrochophore (60 hpf-66 hpf)

Diagnostic feature: Parapodia visible but cannot move yet. The chaetae of the third chaetigerous segment reach the posterior end of the larva (scheme: Figure 3).

**Figure 21:**
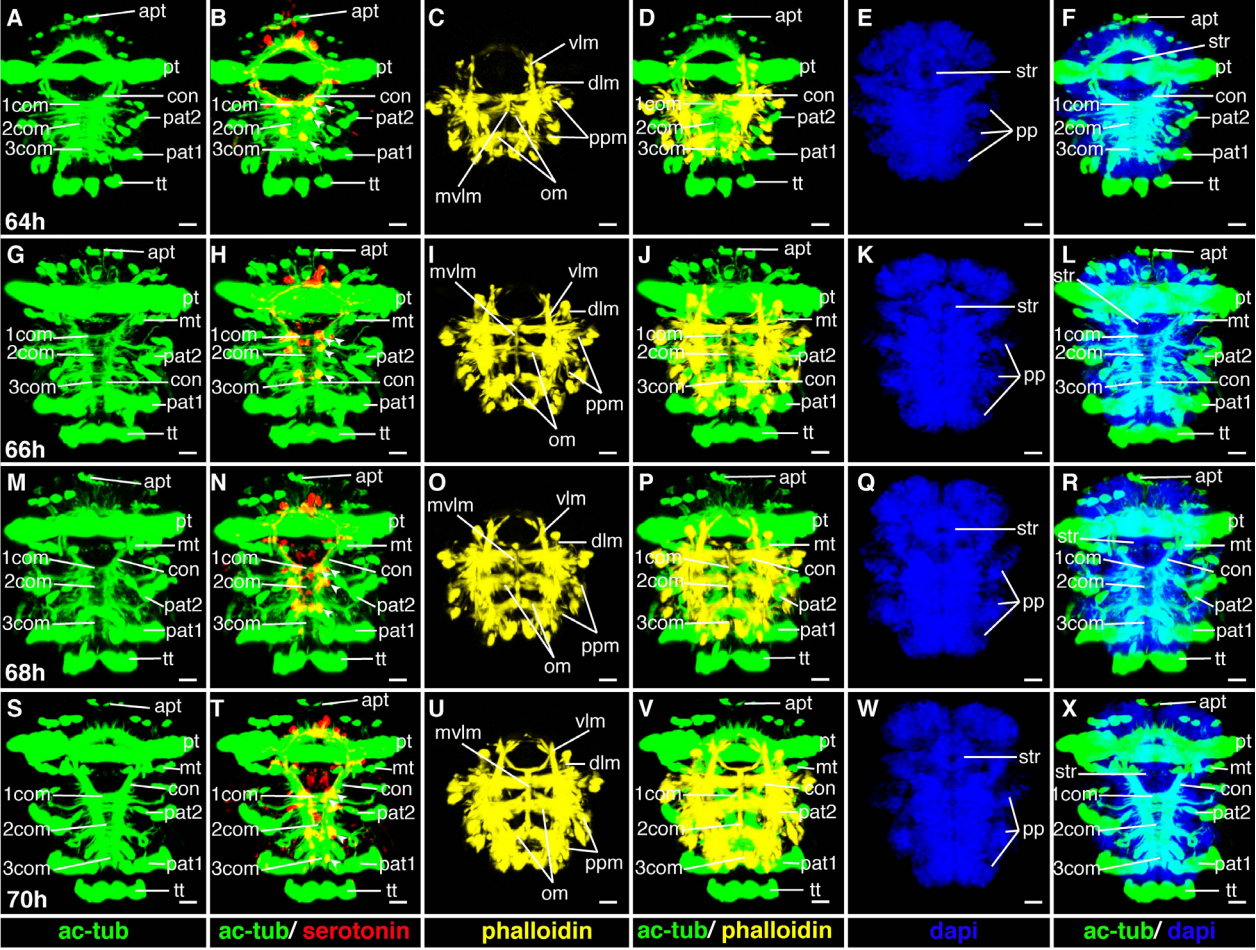
**Ventral nerve cord and muscle development of *P. dumerilii*, 64 - 70 hpf, ventral view, anterior up**. The age of the larvae in each row is given in the lower left corner of the first picture of each row. The displayed staining is indicated at the bottom of each column. A, G, M, S: The connectives (con) and commissures (com) are intensely stained. The connectives have a band-like shape. B, H, N, T: Additional serotonergic cells develop in the ventral nerve cord (white arrow heads), in addition to the unpaired serotonergic cell (white star). C, D, I, J, O, P, U, V: Parapodial muscles (ppm) and oblique muscles (om) as well as the unpaired medial ventral longitudinal muscle (mvlm) are well visible. E, K, Q, W: The metatroch (mt) becomes visible posterior to the prototroch (pt) around 66 hpf. F, L, R, X: The parapodia (pp) grow in size. CLSM microscopy, maximum projection, Imaris surpass mode. Scale bar in all images 20 μm. Further abbreviations see abbreviations list.

The beginning of this stage is marked by the formation of the parapodia. These become bulky and clearly visible (Figure 18[F, J, N], Figure 19[Q, W], and Figure 21[E]). In addition, the chaetae of the third chaetigerous segment grow rapidly and expand to the posterior end of the larva (Figure 18[F, J, N, R]). They ultimately reach the same length as those of the first and second chaetigerous segments. The trunk continues to elongate (Figure 18[F, G, J, K, N, O, R, S]). This causes a change in the overall body shape of the larva from conical to more torpedo-like and slender (Figure 18[F, G, J, K, N, O, R, S]).

**Figure 22:**
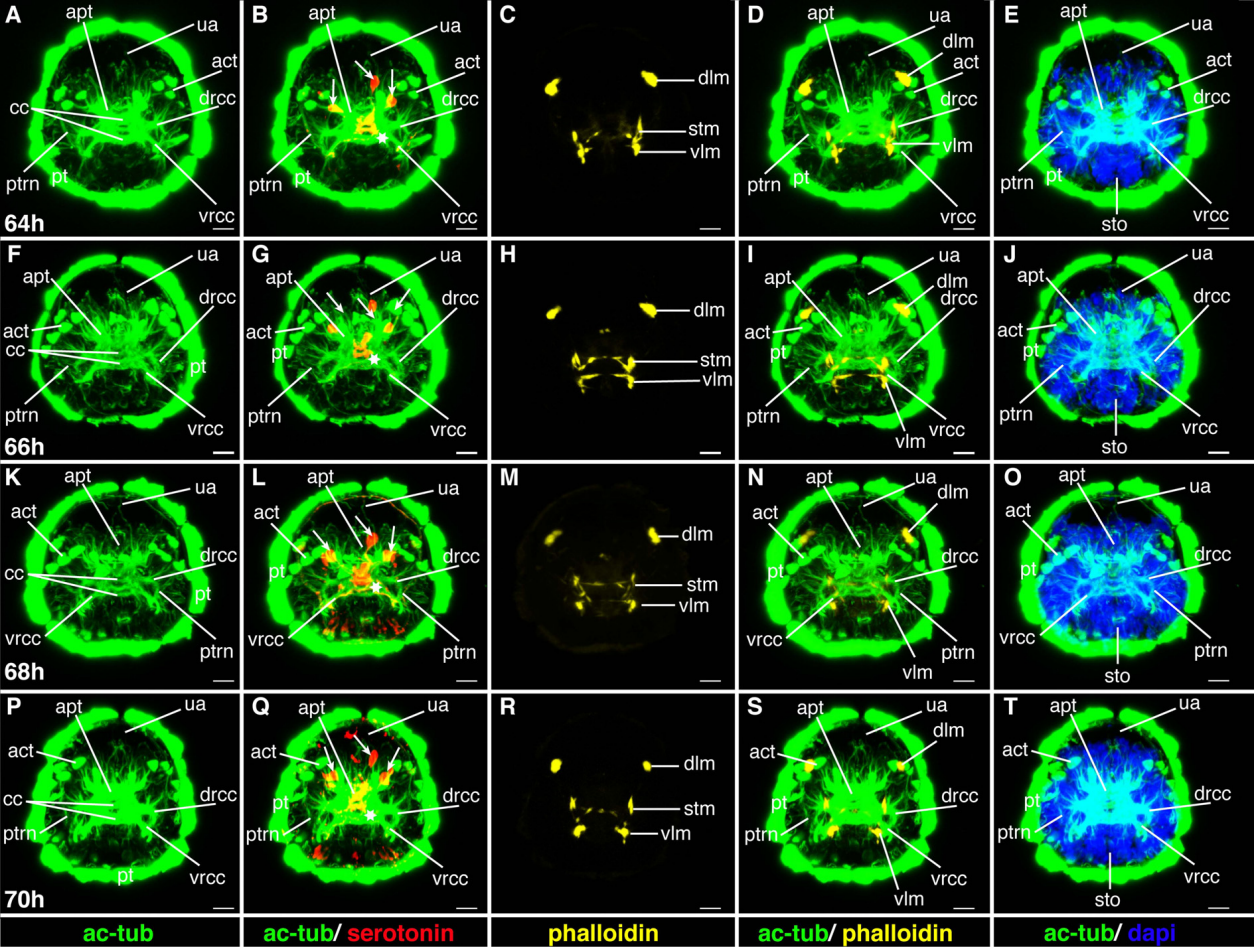
**Brain and muscle development of *P. dumerilii*, 64-70 hpf, apical view, dorsal side up**. The age of the larvae in each row is given in the lower left corner of the first picture of each row. The displayed staining is indicated at the bottom of each column. A, F, K, P: The dorsal and ventral branches of the circumesophageal connectives (drcc and vrcc) continue to approach each other. The distance between the prototroch ring nerve (ptrn) and the prototroch (pt) increases further (e.g. compare A, F, K and P). Therefore, the overall appearance of the brain is more compact. B, G, L, Q: As it was the case during earlier time points, four serotonergic cells are visible in the brain (the central one marked with a white star, the more dorsal ones marked with white arrows). C, D, H, I, M, N, R, S: Musculature around the stomodeum (stm) forms an arch, which branch off the ventral longitudinal muscles (vlm). E, J, O, T: Additional ciliated cells become visible in the akrotroch to form a line on either side of the dorsal head. CLSM microscopy, maximum projection, Imaris surpass mode. Scale bar in all images 20 μm. Further abbreviations see abbreviations list.

During this stage, the formation of the akrotroch starts. The akrotroch comprises a row of ciliated cells anterior to the prototroch. It begins to form in a position dorsal to the apical tuft, and expands ventro-laterally, just ventrally of the adult eyes, towards the prototroch (Figure 20[K, L, N, O-Q, S, T], and Figure 22[A, B, D, E]).

The stomodeum, or larval foregut, invaginates further into the head and elongates along the anterior-posterior axis. Due to the elongation, the stomodeal opening becomes slit-like (Figure 19[W, X], and Figure 21[E, F]). This process continues throughout the following stages.

#### Characteristic features in the nervous system and musculature

The brain and commissures in the ventral nerve cord show an increasing density of neurites as apparent from more intense anti acetylated alpha-tubulin staining (cf Figures 19, 20, 21 and 22). The ventral medial longitudinal muscle elongates further until it reaches the posterior border of the third chaetigerous segment (Figure 19[O, P, U, V], and Figure 21[G, D]).

### Nectochaete larvae

Three larval chaetigerous segments fully developed with ciliary bands, parapodia and chaetae. Non-feeding. Mixed pelago-benthic lifestyle. Bilateral arrangement of akrotroch, metatroch and paratrochs allows ciliary swimming in straight lines without rotation. During swimming, parapodia and chaetae are either laid flat along the body in order to streamline, or extended outward in order to break. These movements occur synchronously in right and left body halves. In contrast, parapodia and chaetae are moved out of phase in right and left body halves for benthic crawling. Development remains synchronous.

### Early nectochaete (66 hpf-75 hpf)

Diagnostic feature: Parapodia start moving independently. Formation of the metatroch. Akrotroch fully developed. Rapid elongation of the trunk. Antennae not visible yet (scheme: Figure 3).

At this stage, the larvae begin to show some occasional crawling on the substrate using their parapodia, which they can move independently due to the well-developed parapodial muscles. Nevertheless, most of the time they continue to swim with their ciliated bands.

**Figure 23:**
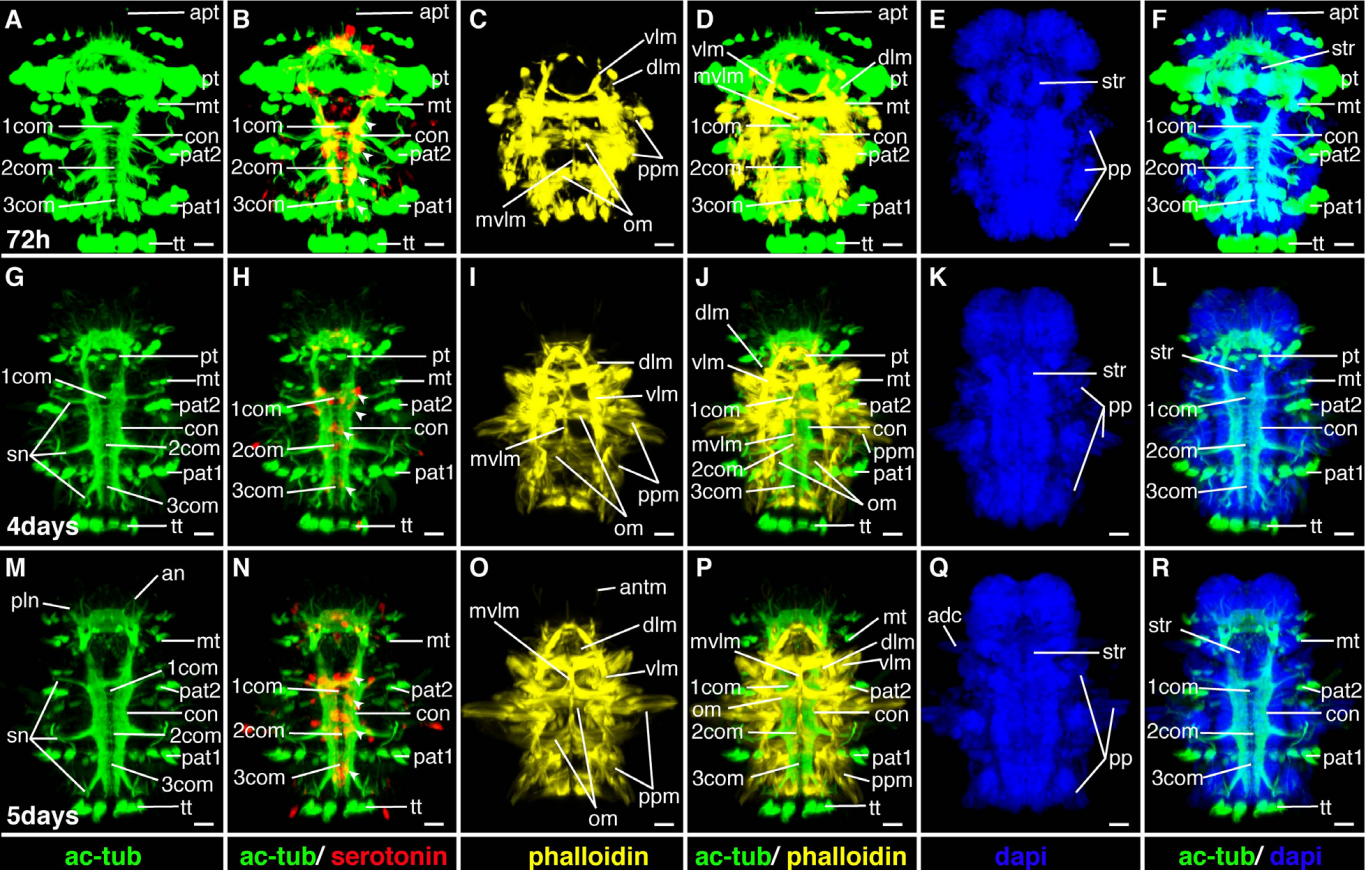
**Ventral nerve cord and muscle development of *P. dumerilii*, 72 hpf - 5 days post fertilization, ventral view, anterior up**. The age of the larvae in each row is given in the lower left corner of the first picture of each row. The displayed staining is indicated at the bottom of each column. A, G, M: The ventral nerve cord is well visible. The connectives become broader. Therefore, the commissures appear shorter than in previous stages. The segmental nerves (sn) become well visible. Antennal nerves (an) and palpi nerves (pln) are visible. B, H, N: More serotonergic cells develop in the ventral nerve cord (white arrow heads). The unpaired serotonergic cell, which was marked by a white star in figure 7, 10, 11, 13, 15, 18, 21, 19, 26 and 28 is not visible anymore. C, D, I, J, O, P: Parapodial muscles (ppm) and oblique muscles (om) as well as the unpaired medial ventral longitudinal muscle (mvlm) are well visible and a complex set of muscles develop in the head region, e.g. the antennal muscles (antm). E, K, Q: The dorsal branch of the anterior cirri (adc) begins to elongate. CLSM microscopy, maximum projection, Imaris surpass mode. Scale bar in all images 20 μm. Further abbreviations see abbreviations list.

**Figure 24:**
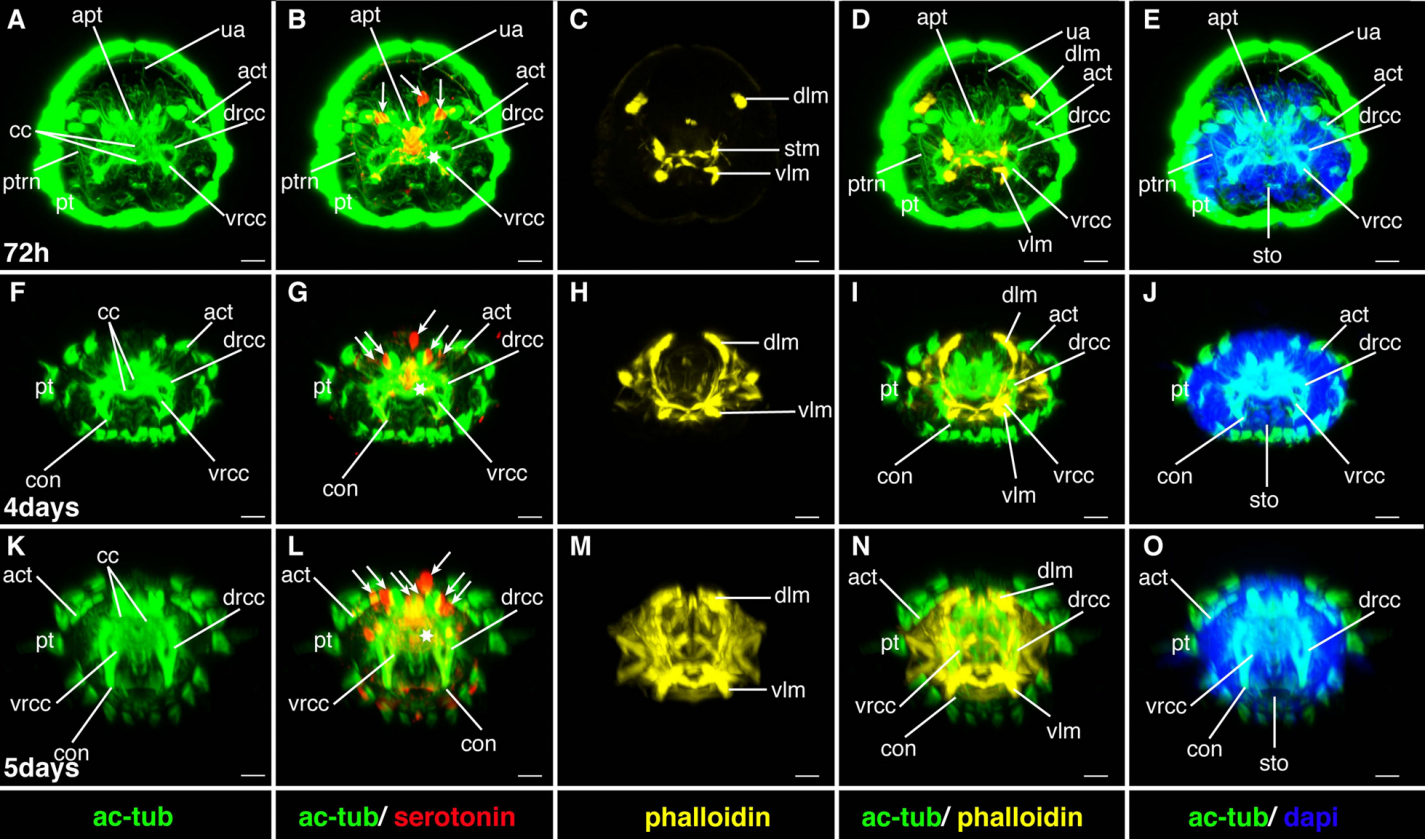
**Brain and muscle development of *P. dumerilii*, 72 hpf - 5 days post fertilization, apical view, dorsal side up**. The age of the larvae in each row is given in the lower left corner of the first picture of each row. The displayed staining is indicated at the bottom of each column. A, E, F, J, K, O: The dorsal and ventral branches of the circumesophageal connectives (drcc and vrcc) continue to approach each other. Finally only a small gap remains between the dorsal and ventral branch of the circumesophageal connectives at 5 days post fertilization (L). The prototroch ring nerve (ptrn) and the dorsal unpaired axon (ua) cannot be distinguished anymore from 5 days post fertilization onwards with the methods used in this study (L). B, G, L: New serotonergic cells become visible in the dorsal part of the brain at 4 and 5 days post fertilization (white arrow heads), additionally to those serotonergic cells that were present during previous stages (the central one marked with a white star, the more dorsal ones marked with white arrows). C, D, I, M, N: A complex pattern of muscles develops inside the head. CLSM microscopy, maximum projection, Imaris surpass mode. Scale bar in all images 20 μm. Further abbreviations see abbreviations list.

Below the prototroch, an additional row of cilia appears on the ventral side, the metatroch (Figure 18[T], Figure 21[G, H, J, L-N, P, R-T, V, X], and Figure 23[A, B, D, F]). It does not extend all the way around the head but leaves a gap where the stomodeum is located, and fuses with the prototroch on the lateral sides (Figure 21[G, H, J, L-N, P, R-T, V, X], and Figure 23[A, B, D, F]). The formation of the akrotroch is completed at the beginning of this stage. It comprises four ciliated cells on each side (Figure 22[F, G, I, J, K-N, O-R, T], and Figure 24[A, B, D, E]).

**Figure 25:**
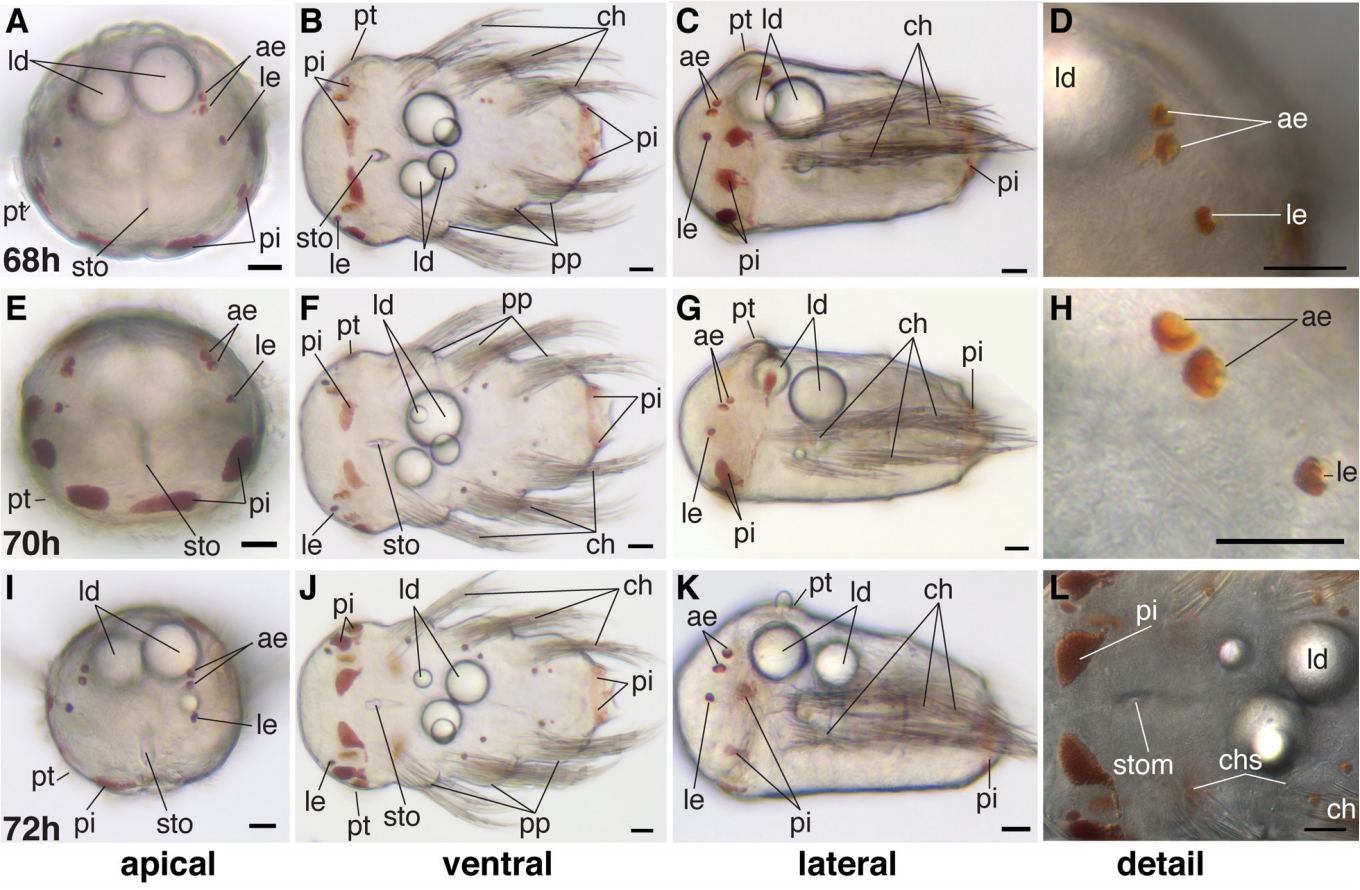
**Series of conventional light microscopy images of *P. dumerilii*, 68-72 hpf**. The arrangement of images is similar to figure 10. B, F, J: The trunk of the larvae elongates rapidly. D, H: The pigmentation in the adult eyes (ae) increases. C, G, K: The lipid droplets (ld) start to move posteriorly. B, F, J: The stomodeum (sto) appears slit-like due its invagination. Scale bar in all images 20 μm. Further abbreviations see abbreviations list.

Trunk elongation proceeds rapidly during this stage. At 66 h the early nectochaete larvae are around 1.5-times longer than wide at the level of the prototroch, whereas at the end of this stage the length is twice the width (Figure 18[R, S], and Figure 25[B, C, F, G, J, K]).

The elongation of the stomodeum along the anterior-posterior axis continues (Figure 18[R], and Figure 25[B, F, J]). The anlage of the proctodeum (hindgut opening) becomes visible. During this stage, it is composed of a small group of cells posterior to the macromeres (data not shown).

The adult eyes develop much stronger pigmentation and approach each other. The larval eyes persist and remain clearly visible (Figure 18[Q, S], and Figure 25[A, C-F, H, I, K]).

Up to this point, the two dorsal lipid droplets have remained slightly anterior to the prototroch or at prototroch level. The ventral lipid droplets (which are usually a bit smaller than the dorsal ones) remain positioned at the level of the first chaetigerous segment. Yet, at the end of this stage, the lipid droplets move posteriorly, the ventral droplets reaching the posterior border of the first chaetigerous segment and the dorsal droplets that of the second chaetigerous segment (Figure 18[R, S], and Figure 25[B, C, F, G, J, K]).

In addition to the pigmented regions mentioned for the previous stages, early nectochaete larvae develop 1-2 pigmented spots at the base of each parapodium (Figure 18[R, S], and Figure 25[B, C, F, G, J, K]).

#### Characteristic features in the nervous system and musculature

The dorsal and ventral roots of the circumesophageal connectives continue to approach each other (Figure 22[A, B, D, F-H, K, L, N, P, Q, R], and Figure 24[A, B, D]). The connectives and commissures in the ventral nerve cord appear thicker. The connectives develop a band-like shape (Figure 21[G, H, J, L-N, P, R-T, V, X], and Figure 23[A, B, D, F]). Additional serotonergic cells develop in the ventral nerve cord (Figure 21[H, N, T], and Figure 23[B]). The musculature around the stomodeum develops and an arch of muscles, which branch off the ventral longitudinal muscles, forms anterior to the stomodeum (Figure 21[C, D], and Figure 22[H, I, M, N, R, S]). Additional muscle fibers are added to the trunk muscles, which conveys them a thicker appearance and more intense phalloidin staining (Figure 21[I, J, O, P, U, V], and Figure 23[C, D]).

### Mid-nectochaete (75 hpf- 4 dpf)

Diagnostic feature: Formation of the antero-dorsal pair of tentacular cirri, anal cirri and antennal stubs (scheme: Figure 3).

**Figure 26:**
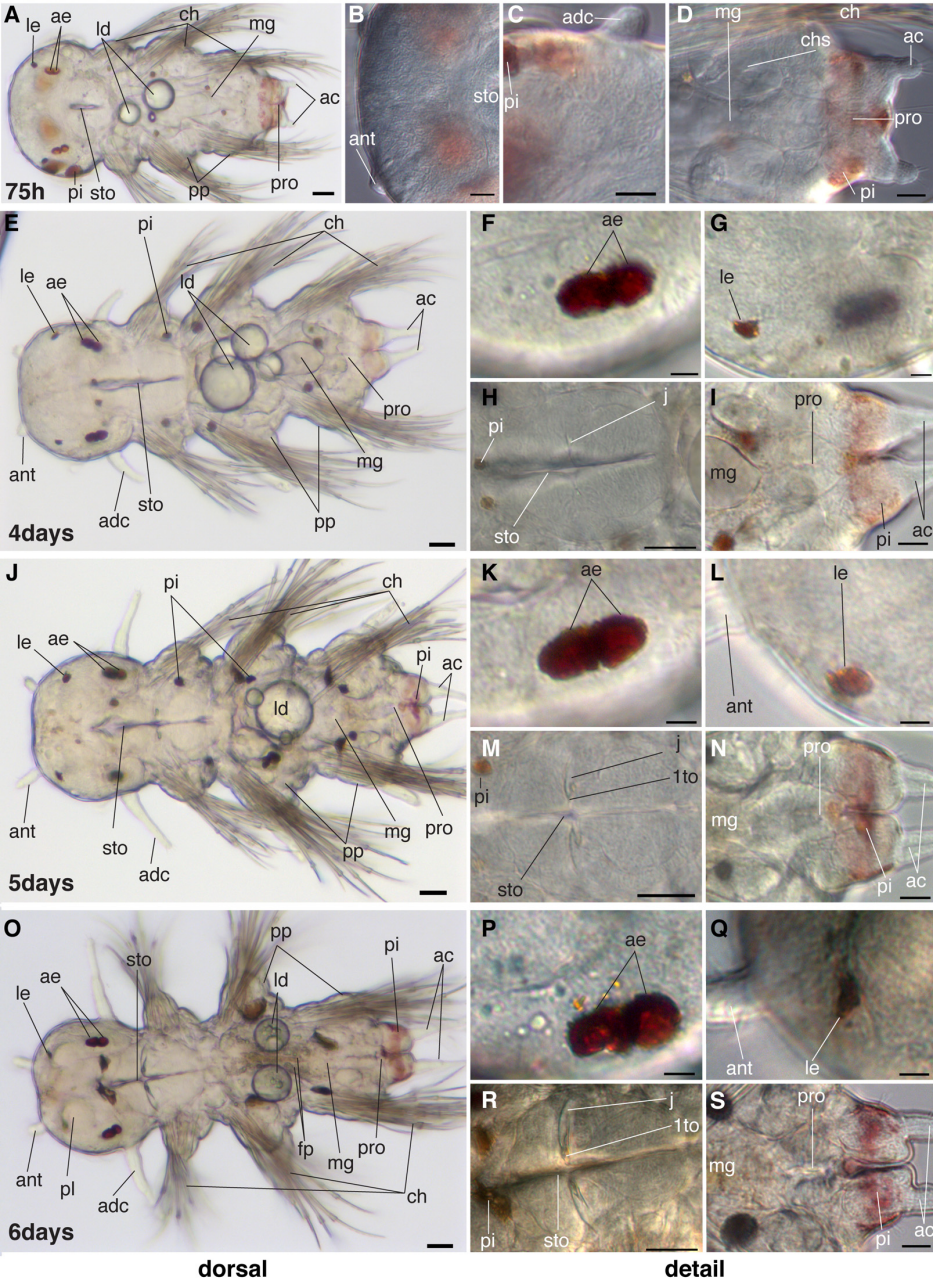
**Series of conventional light microscopy images of *P. dumerilii*, 75 hpf (mid nectochaete) - late nectochaete**. A-D: larvae at 75 hpf. A: Ventral view of larvae, anterior side left. B: Detail of the developing antennae (ant). C: Detail of the developing anterior dorsal cirrus (adc). D: Detail of the developing anal cirri and the forming midgut (mg) and proctodeum (proc). E, J and O: Dorsal view of larvae, anterior side left. The age is indicated in the lower left corner of each picture. The antennae (ant), anterior dorsal cirri (adc) and anal cirri (ac) increase in length. The lipid droplets (ld) get absorbed. F-I, K-N and P-S: Several details of larvae at 4 days, 5 days and 6 days respectively. Compare A, F, K and P: The adult eyes (ae) increase in size compared to previous stages. H, M, R: The jaw (j) is increasing in size and the primary tooth (1to) becomes well visible. I, N, S: The proctodeum (proc) forms a connection to the midgut (mg) at around 5 days. O: Food particles (fp) are clearly visible in the midgut (mg). The palpi (pl) are visible as circular structures. G, L, Q: The larval eyes are remaining. Scale bar in all images 20 μm. Further abbreviations see abbreviations list.

Characteristic for this stage is the outgrowth of sets of appendages likely to function as sense organs: the antero-dorsal pair of tentacular cirri and the anal cirri (Figure 26[B-E, I]). The antennae, located at the anterior tip of the head, are only visible as small stubs (Figure 26[B]).

Lateral to the stomodeum, the antero-dorsal pair of tentacular cirri grows out, equally long and slender (Figure 26[C, E]). Likewise, the anal cirri start to grow at the pygidium (Figure 26[A, D, E, I]).

The whole larva continues to grow in length, and the body shape changes from torpedo-like to worm-like (Figure 26[A, E]). The head can be clearly distinguished from the trunk due to a constriction that forms between the head and the first chaetigerous segment (Figure 26[A, E]).

The larval eyes are still present (Figure 26[A, G]). The adult eyes grow in size. The two pairs of adult eyes on each side are now so closely spaced that they are separated only by a medial constriction (Figure 26[A, E, F]).

The pigmented spots at the base of the parapodia increase in size and additional pigment in the head region appears (Figure 26[E, H]).

The stomodeum/foregut shows pronounced elongation. It exhibits well-developed lips and becomes surrounded by an additional circular layer of cells. Embedded in the larval foregut, the jaws begin to form (Figure 26[A, E]). Initially, a single, small tooth is visible on each side and forms the pointy anterior end of the jaw, also known as the primary tooth (Figure 26[H]).

The macromeres start cellularization, thus initiating formation of the midgut epithelium. During midgut formation, the two ventral lipid droplets move further posteriorly into the second chaetigerous segment (Figure 26[A, E]). The proctodeum becomes cone-shaped, with the broad side forming a connection with the midgut, and the narrow side forming the anal lumen between the anal cirri in the pygidium (Figure 26[A, E, I]). Towards the end of this stage, the stomodeum establishes contact with the midgut and subsequently, the proctodeum connects to the midgut [37]. The digestive tract is fully formed.

#### Characteristic features in the nervous system and musculature

The brain grows noticeably (Figure 24[F, G, I]). Additional serotonergic cells develop in the brain (Figure 24[G]). In the trunk, the segmental nerves are now clearly visible (Figure 23[G, H, J, L, M]). In addition, muscles and nerves begin to develop in conjunction with the developing antennae, tentacular cirri. The stomodeum establishes contact with the midgut. Subsequently, the proctodeum connects to the midgut [37] and the digestive tract is fully formed (Figure 23[G-I]).

### Late nectochaete (5 days - 7 days)

Diagnostic feature: antennae elongate, palpi become visible, beginning of food intake (scheme Figure 4).

The antennae, previously visible as small stubs only, grow out and become long and slender (Figure 26[J, O]). The pair of palpi, likely to function as chemosensory organs, forms on both sides of the mouth opening. They appear as circular, slightly bulky structures (Figure 26[O]).

The gut becomes functional and the larvae begin to feed on algae and detritus. Since, the larvae are transparent, food is visible in the gut (Figure 26[O]). However, at this stage, the midgut lumen is only slit-like. The lipid droplets, utilized as a food source at earlier stages begin to be resorbed to variable degree (Figure 26[J, O]). Therefore, the number of droplets may differ among individuals.

The jaws grow rapidly (Figure 26[M, R]). The larval eyes are still present (Figure 26[L, Q]).

During this stage, the transition progresses from pelago-benthic to fully benthic lifestyle. The late nectochaete larvae are mainly found crawling on the substrate and less frequently swimming in the water column using their ciliary bands.

The end of this stage is not reached synchronously but differs among individuals.

#### Characteristic features in the nervous system and musculature

The brain continues to grow rapidly (Figure 24[K]). Two additional serotonergic cells develop in the brain (Figure 24[L]). The musculature around the stomodeum increases in complexity and a basket of muscles develops around the jaws to form the pharynx (Figure 23[O, P], and Figure 24[M, N]). Muscles and nerves, which are associated with the developing antennae, tentacular cirri, palpi and anal cirri increase in length (Figure 23[M, O, P]).

##### Errant juvenile stages (from settlement metamorphosis to cephalic metamorphosis)

Benthic stages following larval settlement. Fully feeding worms with 3 to 5 chaetigerous segments, which freely move around by undulatory crawling. The fourth and fifth chaetigerous segment form by terminal addition from a posterior growth zone. First stages with flexible timing depending on food supply.

##### 3-segmented errant juvenile (from the first filled gut until the fourth chaetigerous segment is fully formed)

Diagnostic feature: No lipid droplets visible in the gut. Barrel-shaped midgut filled with food. Settlement metamorphosis completed during this stage. Growth of the fourth chaetigerous segment (scheme: Figure 4).

**Figure 27:**
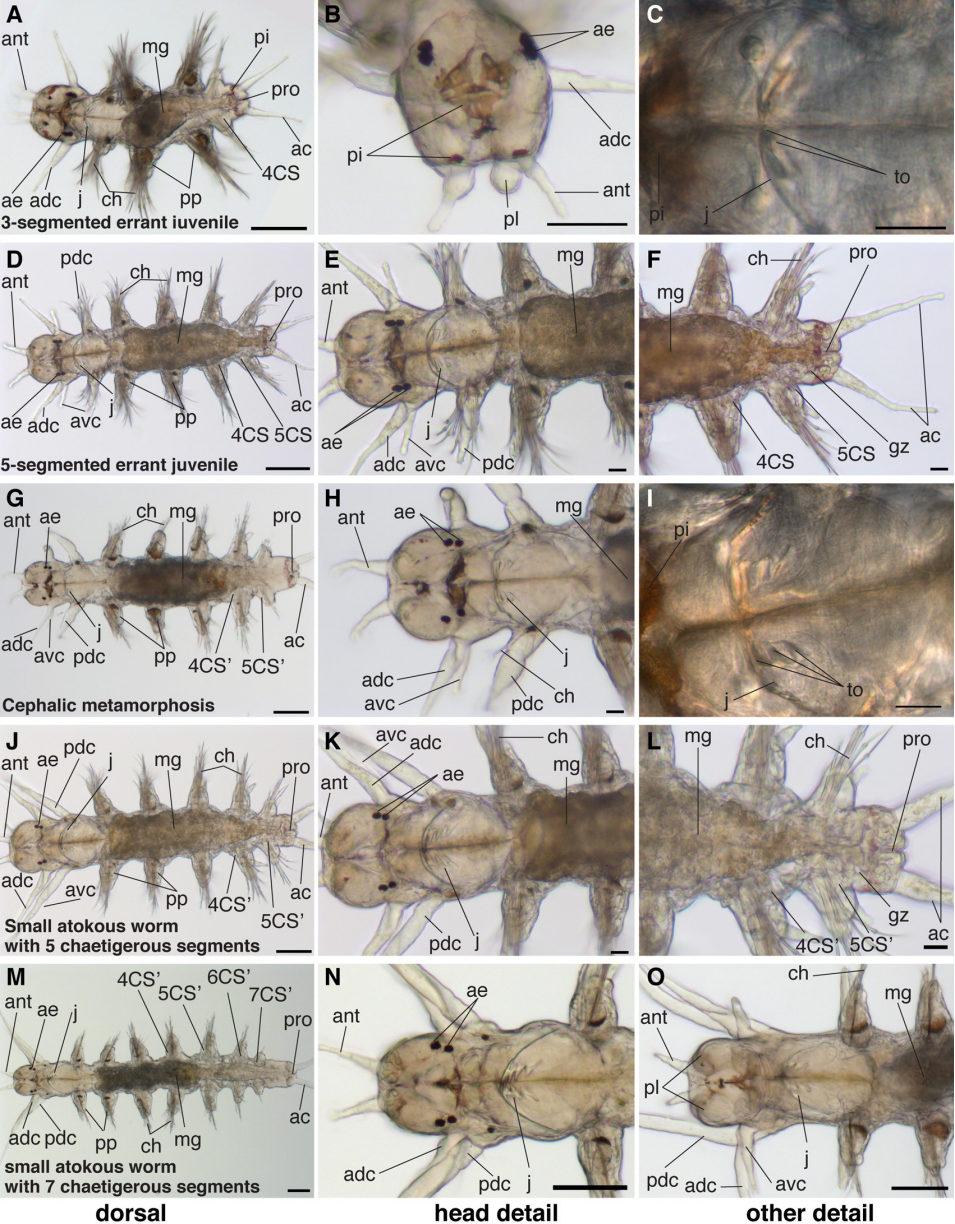
**Series of conventional light microscopy images of *P. dumerilii*, 3-segmented errant juvenile - small atokous worm with 7 chaetigerous segments**. The stage of the juvenile in each row is given in the lower left corner of the first picture of each row. In order to give an overview on the morphological changes throughout development, for each time point a dorsal view, anterior side left (first column: A, D, G, J, M), a close-up of the head (second column: B, E, H, K, N) and several details (third column: C, F, I, L, O) are shown. B: Anterior view of juvenile, dorsal side up. E, H, K and N: Dorsal view of juvenile, anterior side left. Compare A, E, H, K and N: Note the transformation of the most anterior pair of parapodia into the dorsal pair of posterior cirri (pdc). While the number of segments is indicated with CS4, CS5 respectively before cephalic metamorphosis, the numbers are indicated with CS4', CS5' after cephalic metamorphosis. Please note, that CS5 and CS5' are not the same segment. A more detailed description of the cephalic metamorphosis is given in the text. C and I: Stomodeum at a higher magnification, the jaws (j) increase in size and additional teeth (th) form. F and L: All secondary segments are formed by the posterior growth zone (gz). Scale bar in A, D, G, J, M: 100 μm, in all other images 20 μm. Further abbreviations see abbreviations list.

By the beginning of this stage, the lipid droplets are totally resorbed (Figure 27[A]). The gut is well developed and wide and fills almost the entire trunk. It becomes narrower toward the hindgut. When enough food is available, the gut is completely filled with food particles. In the absence of food, the juveniles become cannibalistic. The jaws are rapidly increasing in size and additional teeth are added (Figure 27[C]).

During this stage, the settlement metamorphosis, which started during the late nectochaete stage, is completed. It is characterized by the loss of larva-specific structures such as the prototroch and the apical tuft (Table 1).

**Table 1:** Summary of the metamorphoses of P. dumerilii.

	lifestyle before metamorphosis	lifestyle after metamorphosis	morphological changes
**Settlement metamorphosis**	planktonic	errant	digestive tract becomes functional, ciliary bands start to get abolished, apical tuft is lost

**Cephalic metamorphosis**	errant	tubicolous	transformation of the first pair of parapodia, formation of tubes, larval eyes and ciliary bands disappear

**Sexual metamorphosis**	tubicolous	pelagic	maturation of gametes, enlarging adult eyes, development of paddle shaped chaetae and epitokous musculature

Overview of the metamorphoses of *P. dumerilii *showing the lifestyle before and after metamorphosis, as well the corresponding morphological changes.

The palpi elongate slowly (Figure 27[B]). The antennae double their length with respect to the previous stage (Figure 27[A, B]). The antero-dorsal tentacular cirri elongate rapidly, and point in an antero-lateral direction (Figure 27[A, B]). The anal cirri also grow in length (Figure 27[A]).

Towards the end of this stage, the fourth chaetigerous segment is growing (Figure 27[A]) representing the first segment proliferated from the posterior growth zone.

The errant juveniles develop spinning glands in their parapodia and start to form first mucus toward the end of this stage. Initially, they do not form complete tubes but may form orderless networks [43,51].

The loss of synchrony between individuals of the same batch and age becomes more and more pronounced over time. As a result, within one and the same batch some individuals may have a fully formed fourth segment and thus reach the following stage, while in others the fourth segment just begins to form. Therefore, not only the beginning but also the time-span required to reach the end of this stage differs considerably even among individuals living in the same container dish. As a rule of thumb, a fully formed fourth segment is present by approximately two weeks of development at 18°C.

##### 4- and 5-segmented errant juveniles (completion of fourth chaetigerous segment to cephalic metamorphosis)

Diagnostic feature: fourth chaetigerous body segment fully formed; fifth chaetigerous segment growing or fully formed (scheme: Figure 4).

During thus stage (Figure 27[D-F]), the jaws continue to grow rapidly and additional teeth are added (Figure 27[E]). A second set of tentacular cirri develops at the head - the antero-ventral tentacular cirri. Together with the antero-dorsal tentacular cirri (see above), they belong to the cryptic segment. The antero-ventral tentacular cirri rapidly increase in length (Figure 27[D, E]).

##### Cephalic metamorphosis (from the beginning to the end of cephalic metamorphosis)

Diagnostic feature: loss of chaetae at the first pair of parapodia, which marks the beginning transformation of the first pair of parapodia into the posterior pair of tentacular cirri. Cephalization (scheme: Figure 5).

**Figure 28:**
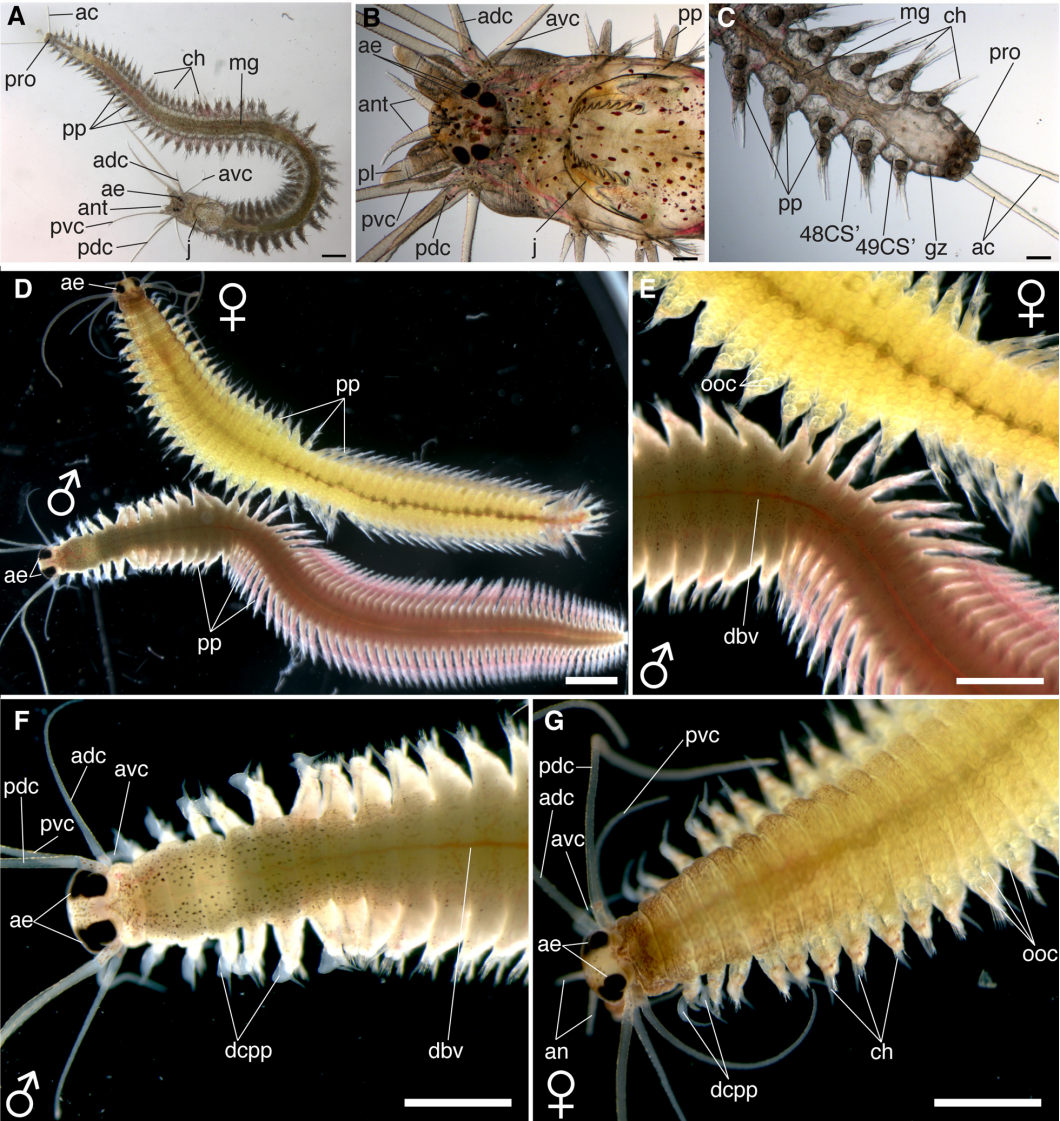
**Series of conventional light microscopy images of *P. dumerilii*, small atokous worm and heteronereis**. All images: Dorsal view, anterior side left. A: Overview, small atokous worm with 48 chaetigerous segments. B: Head of the same specimen as shown in A, at a higher magnification. The dorsal and ventral branch of the anterior and posterior cirri are visible, as well as the palpi (pl), antennae (ant) and the well developed jaws (j). C: Posterior end of the same specimen at higher magnification, posterior to the last chaetigerous segment is the growth zone (gz) located where new segments are formed. D: Overview, male and female may be similarly sized. Female (♀), top, appears yellowish, due to the oocytes in the coelomic cavity. Male (♂) bottom, appears white due to spermatozoans in the anterior part and red due to dense accessory blood vessels in the posterior part. The parapodia (pp) in the posterior body part are flattened with paddle-like chaetae. E: Detail of the male and female body at a higher magnification. Note the numerous visible oocytes (ooc) inside the female's body and the different shape of the parapodia (pp) in the anterior and posterior body part. F: Anterior part of a mature male. Note the enlarged adult eyes (ae) in comparison to B. The dorsal cirri of the parapodia (dcpp) in segment three to nine are clearly club-shaped. D: Anterior part of a mature female, note also here the enlarged adult eyes (ae) and the oocytes inside the coelomic cavity and the parapodia. The dorsal cirri of the parapodia (dcpp) in segment three to seven are slightly club-shaped. Scale bar in A: 500 μm. Scale bar in B and C: 100 μm. Scale bar in D, E, F and G: 2 mm. Further abbreviations see abbreviations list.

During cephalic metamorphosis, the first pair of parapodia is transformed into the posterior pairs of tentacular cirri and the first chaetigerous segment is incorporated into the head (Table 1 Figure 27[M, P, O]). These processes have been described in great detail by Hempelmann [52] and Hauenschild and Fischer [37] and Fischer [53], and are therefore only briefly summarized here. At the notopodium, which is the dorsal branch of the parapodium, the ventral notopodial lingule begins to elongate (compare Figure 27[E] of previous stage). The sixth chaetigerous segment forms (Figure 27[G]) while the first pair of parapodia has nearly completely lost its chaetae and the notopodial lingule have turned into long and slender structures that point in an anterior-lateral direction (Figure 27[G, H]). It is now located closer to the head and grows, longer than the antero-dorsal pair of tentacular cirri (Figure 27[J, K]). By the end of cephalic metamorphosis, all tentacular cirri point in an anterior-lateral direction (Figure 27[J, K]) [51,54]. During the transformation of the first parapodium, the entire segment changes shape and is added to the head (Figure 27[D, E, G, H, J, K, M, N]). Later, the postero-ventral cirrus forms at the posterior pair of tentacular cirri. Four cirri in total are visible on each side (Figure 28[B]).

Using their spinning glands, the animals start to build their characteristic tubes on the bottom or in the corners of the tank. The formation of the tubes by the spinning glands in the parapodia is described in detail by Daly [55]. The young worms leave their tubes only occasionally in case of stress, when they want to relocate or, preferably at night. They swim with undulatory body movements.

The larval eyes seem to disappear during cephalic metamorphosis [37]. The palpi and antennae elongate only slowly while the anal cirri continue to grow rapidly in length (Figure 27[D-H, J-L]).

The midgut elongates rapidly with the elongating body and spans the distance between the second and the second-to-last chaetigerous segment (Figure 27[D, G, L, M]). The proctodeum is positioned in the last segment and in the posterior growth zone (Figure 27[F, L]). Both parts of the gut can easily be distinguished as the midgut is very wide and fills almost the entire segment, whereas the proctodeum is much more slender (Figure 27[F, L]). The jaws grow in size and additional teeth are added (Figure 27[I]). Inside the stomodeum, the pharynx develops into an eversible proboscis, which is heavily muscularized (data not shown).

### Tubicolous juvenile stages

#### Small atokous worm (from completion of cephalic metamorphosis to fully grown adult)

Diagnostic feature: cephalic metamorphosis is finished and the posterior pairs of tentacular cirri are formed. The posterior growth zone buds of a series of further segments. Less than fifty segments (scheme: Figure 5).

The processes following the cephalic metamorphosis are described by Hempelmann [52], Hauenschild [56] and summarized by Hauenschild and Fischer [37], and Fischer [51]. They are only briefly summarized here: Additional segments are successively formed by the posterior growth zone (Figure 28[A-C]). The growth rate increases remarkably and up to one segment per day can be formed [37]. More anterior segments grow in size, thus leading to an increase in the diameter of the juvenile. The jaws continue to grow until they ultimately contain one primary and nine secondary teeth (Figure 28[B]).

The young worms leave their tubes only occasionally in case of stress, when they want to relocate or in search for food, preferably at night. They swim with undulatory body movements.

### Large atokous worm

Diagnostic feature: tubicolous worm with more than 50 segments. Gametes visible inside body.

The sexually immature atokous worms start to produce gametes in the coelom when they have approximately 50 segments. At this stage, their growth rate slows until they possess around 70 segments and the gametes start to mature inside the body cavity.

### Heteronereis stages

Sexually dimorphic worms: development of yellow females and red-whitish males.

#### Sexual metamorphosis (from the stop of food uptake until animals are ready to leave their tubes and start swarming)

Diagnostic feature: Animals stop food uptake, increase their eye size, subdivide their trunk into two parts with different shapes of parapodia and change body color. The worms still remain in their tubes.

A decrease in the titer of a hormone produced in the neuro-secretory brain centers leads to a profound transformation of the tissues and of morphology. This process may be called "sexual metamorphosis" (Table 1) since it leads from the immature, benthic atokous condition to one which is drastically modified both morphologically and physiologically into a pelagic, sexually mature epitokous form e.g. [51]. The lifespan of *P. dumerilii *in culture from fertilization until maturity is at least three, but on average six to seven months at 18°C, and can take up to 18 months.

A detailed description of the changes during the sexual metamorphosis into the so-called heteronereis or mature adult is given by Hauenschild and Fischer [37], Fischer [51] and Fischer and Dorresteijn [6], and is briefly summarized here (scheme: Figure 6).

The first visible indication of the onset of sexual metamorphosis is the cessation of feeding, which leads to an empty gut three to six days before the animals mature. Later the gut collapses and degenerates to some extend [6]. The dorsal cirri become clearly club-shaped at the parapodia of the first seven segments in the maturing males, while they become only slightly club-shaped in the maturing females (Figure 28[F, G]). Later, the parapodia of the posterior two-thirds of the male body flatten and develop paddle-like chaetae, which are used for fast swimming (Figure 28[E]).

The eyes increase in size and a part of the chromatophores degenerates inside the body (Figure 28[F, G]). The developing gametes become visible through the body wall (Figure 28[E]). While the oocytes are yellow, which contributes to the yellow color of the maturing females, the mass of spermatozoans appear white and cause the white color of the anterior part of the male body (Figure 28[D, E, F, G]). The posterior part of the males turns red as a result of the large number of accessory blood capillaries (Figure 28[D, E]).

Major parts of the musculature degenerate and a new epitokous muscle type for rapid swimming develops, in large parts through the transdifferentiation of pre-existing atokous fibers.

#### Sexually mature adult/heteronereis (from the beginning of swarming to death)

Diagnostic feature: Rapid swimming in straight lines. Nuptial dance.

Males are mature for slightly longer than one day, females only for a few hours, synchronized by lunar periodicity (e.g. [57]). Finally, the mature animals become pelagic. They swim rapidly searching for, and together with, other mature individuals. Males and females attract each other by pheromones [58,59]. This behavior is called swarming and ends in the nuptial dance, when males and females rapidly swim in a circle. The females deliver the eggs through disruptions/fissures between the segments, while the males deliver the sperm through a number of newly formed papillae at the posterior end. While males and females deliver the gametes they are swimming in close circles around each other [51]. The eggs are fertilized in the water.

After spawning, males and females die.

#### Development at different temperatures in comparison with 18°C

**Figure 29:**
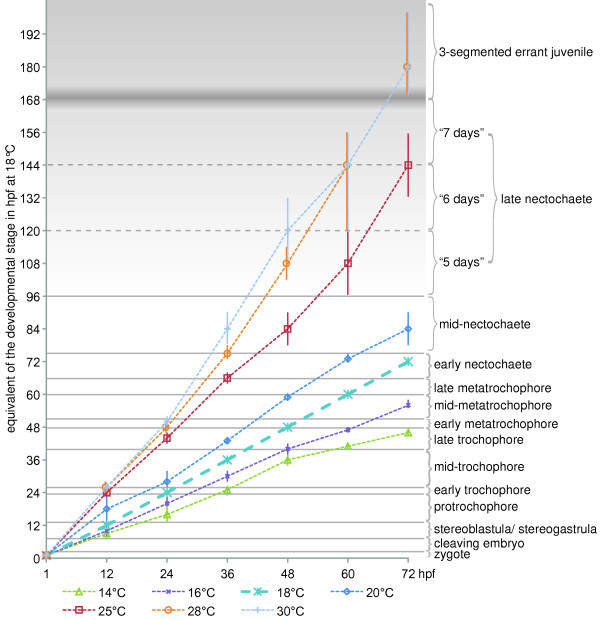
**Influence of temperature on developmental speed in *P. dumerilii***. Diagram, which shows the developmental stage reach 12 h, 24 h, 36 h, 48 h, 60 h and 72 h post fertilization, growing at 14°C, 16°C, 18°C, 20°C, 25°C, 28°C and 30°C. Sampling was performed every 12 h for 72 h. The error bars indicate the range within the stage, at which the embryos or larvae were found. Within this range it was not possible to determine the stage more precisely. The gradient in the chart background indicates the decreasing synchrony from the late nectochaete stage onwards. The transversal lines indicate the end of one stage and the beginning of the following one, The duration of the late nectochaete stage and the beginning of the following stage vary, indicated by the bold blurred line.

*P. dumerilii *developmental speed is obviously temperature-dependent [6], yet this dependency has never been quantified. To test the effect of different temperatures on the developmental speed of *P. dumerilii*, larvae were raised at: 9°C, 14°C, 16°C, 20°C, 23°C, 25°C, 28°C, 30°C and 34°C. While the eggs did not develop at 34°C and embryos did not develop beyond the cleavage stage at 9°C, all other temperatures tested resulted in normally developing larvae. At these permissive temperatures, development accelerates or decelerates almost linearly with temperature when compared to standard (18°C) conditions (Figure 29).

For illustration, after three days of development, when larvae raised at the standard temperature have developed into early nectochaetes, larvae raised at 14°C have only reached the late trochophore stage, while those reached at 28°C have already reached the three segmented errant juvenile stage and started feeding.

### Bioinformatic resources

To facilitate the use of the developmental staging system defined above for electronic annotations we have generated two resources. First, we have created a staging ontology representing the list of developmental stages. Today ontologies are a common standard for annotating biological data [60]. They allow for classification of annotated data and also provide a common language of annotation terms for data analysis. The *P. dumerilii *stage ontology was set up as an OBO formatted file using OBOedit http://oboedit.org/. Each term within this ontology represents a developmental stage, which has a unique name, list of synonyms and a definition. Terms are connected via relations that represent the temporal relationships between the stages. The stage ontology can be used to annotate gene expression patterns or phenotypes and is currently used for and complemented by an ontology for *P. dumerilii *anatomy (in preparation). The ontology is available at http://4dx.embl.de/platy and has been submitted to the obofoundry http://www.obofoundry.org using a unique namespace (PD_ST).

The second resource is a database accessible via web interface http://4dx.embl.de:8080/platy. Here, all data necessary for staging embryos are available. A timeline graphic provides an overview and fast access to the individual stage entry with associated images and a list of criteria for each individual stage. This resource allows biologists anywhere to determine the developmental stage of *P. dumerilii *individuals. In the future, this web page will provide other relevant information concerning the model species such as access to the genome and transcriptome.

## Discussion

### Comparison to pre-existing polychaete staging systems

Polychaete larval development shows extreme diversity [61,62], which is not necessarily reflected by usage of different terminology. This may invoke misleading comparisons between non-corresponding stages and morphologies.

The developmental stages defined here are based on existing terminology used for *P. dumerilii *and for other polychaetes [6,36,37,62]. Häcker [36] distinguished five stages of polychaete development: 1) protrochophore (with a broad preoral ciliated band), 2) trochophore (with a narrow band of long cilia - the prototroch) 3) metatrochophore I (simplest form of a segmented larva) 4) metatrochophore II (parapodia appear but are not yet used for locomotion) and 5) nectochaete (parapodia are the main swimming apparatus). Although the meaning of some of the terms has changed over the years, this gross staging system is still in use today. The term protrochophore has been expanded to include larvae which do not have a broad ciliated belt but are very young trochophores slowly rotating on the substrate (e.g. [44,63,64]) and to atrochal polychaete larvae [62,65]. The distinction between metatrochophore I and II is rarely used now and many authors simply describe one stage called metatrochophore [37,66] or do not describe a metatrochophore stage at all [67-70]. Also, the distinction between the metatrochophore II stage and the nectochaete appears problematic [48].

Here, for the sake of continuity and comparability, we build on Häcker's traditional staging system, although some discrepancies are apparent also for *P. dumerilii*. The term "protrochophore" *sensu *Häcker does not fit the situation found in *P. dumerilii*, because this species never develops a very broad band of short cilia. We nevertheless use this term, as was already done by Dondua *et al. *[44]. Furthermore, a "nectochaete" in *P. dumerilii *differs from Häcker's description in that the larvae do not use the parapodia for active swimming. (They are merely used for navigation while the propelling force for swimming is still generated by the cilia, see above.) However, the "nectochaete" is a commonly used term to describe a three to five day old *P. dumerilii *larva (e.g. [6,31,37]) and is thus retained here.

For more precise and refined staging, a subdivision of the trochophore, metatrochophore and nectochaete stages into three (early, mid and late) sub-stages each is introduced here. We consider these terms meaningful also for scientists less familiar with *P. dumerilii *and decided against a numbering system (as used for example for chicken: [71], marble crayfish: [72], *Chaetopterus: *[45], *Capitella*: [73]) that is more neutral in terminology but less descriptive and thus more difficult to memorize. For later stages, we propose to use the onset of feeding and the number of segments. Segment number is a commonly used, time-independent reference system for polychaetes [54,67,70,74,75]

### *P. dumerilii *development is prototypical for other nereidid species

*P. dumerilii *is a member of the monophyletic nereidids, a group already mentioned in pre-linnean writings [76]. Within the nereidids, adults as well as early developmental stages, larvae and juveniles share many similarities between species [77], such as the cleavage pattern, the development of characteristic ciliary bands, the simultaneous appearance of three chaetigerous segments and the variability of pigmentation. Most nereidids undergo two metamorphoses. The settlement metamorphosis encompasses a transition from a planktonic to an errant life style, while during the cephalic metamorphosis, the first chaetigerous larval segment is transformed into a head segment and the young worms become tubicolous (Table 1) [49].

The cleavage pattern and early development of *Alitta succinea *and *Platynereis megalops *was described by E.B. Wilson [42] in great detail. All other nereidids investigated thereafter show similar patterns (e.g. [5,43,49,78]).

*P. dumerilii *larvae develop a series of ciliary bands: the prototroch, an akrotroch, three paratrochs (the anterior ciliary band, called paratroch is actually a metatroch [23]) and a telotroch. These bands are also characteristic for the larvae of other nereidids [49].

Three chaetigerous segments appear simultaneous in *P. dumerilii *during the late trochophore stage. This is also the case in most other nereidids [48,54]. Few species such as *A. succinea *and *P. megalops *show a pronounced time lag prior to the formation of the third chaetigerous segment [42,49].

The long-standing question whether the metatrochophore and nectochaete larvae possess a "rudimentary" or "cryptic" first larval segment was recently addressed by Steinmetz *et al*. (accepted manuscript), who showed via the combined analysis of marker genes and morphological characters that a fourth larval segment indeed exists, at least in *P. dumerilii*. This segment never develops chaetae but bears the anterior pairs of tentacular cirri. It thus resembles the second larval segment after cephalic metamorphosis, which likewise loses chaetae and forms the posterior pairs of tentacular cirri.

In *P. dumerilii*, not only the eyes but also the prototroch, the telotroch and the base of the parapodia show pigmentation during development. The appearance and intensity of pigment varies between different individuals. Similar observations have been made in *Neanthes fucata *(previously called *Nereis fucata*), *A. succinea *and *P. megalops *[42,49].

### Developmental differences between nereidid larvae owing to diverging larval ecology

Despite the overall similarity of developmental patterns of *P. dumerilii *and other nereidids, some differences are also observed that can be accounted for by differences in larval ecology. One well-investigated example is that of *Platynereis massiliensis*, the putative sister species of *P. dumerilii*. These two species are morphologically so similar that they were initially considered two different morphs of a single species, *Nereis dumerilii *[52]. However, both species show considerable developmental differences that appear to be direct or indirect consequences of a difference in larval lifestyle: while the early trochophore of *P. dumerilii *is pelagic, that of *P. massiliensis *remains in the parental tubes until it has grown several segments [52,56]. It develops through a trochoid stage, which rotates slowly around its axis but does not swim freely [43].

Most profoundly, both *Platynereis *species differ in the amount of deposited yolk. While *P. dumerilii *produces eggs of approximately 160 μm in diameter with 64% yolk, the eggs of *P. massiliensis *measure approximately 280 μm in diameter with 90% yolk, thus are more than 10 times larger. Relating to this, cleavage takes nearly four times longer in *P. massiliensis *than in *P. dumerilii *[43]. *P. massiliensis *reaches the equivalent of an early trochophore stage (24-25 hpf at 18°C in *P. dumerilii*) only after 48 h at 18°C [43]. The small zygotes of *P. dumerilii *also cleave faster than that of other nereidids, so that *P. dumerilii *development can be considered relatively fast within this taxon.

The different amount of yolk deposited in the macromeres also influences later larval stages. While the larvae of *P. dumerilii *acquire a slender torpedo-like shape at the early nectochaete stage, other species with larger macromeres are more oval or spherical in shape with a tubby appearance and less clearly visible parapodia as is the case for *P. massiliensis *[43]. Also, the beginning of food uptake depends on the amount of yolk initially available [49]. *P. dumerilii *starts feeding during the late nectochaete stage when only three chaetigerous segments are present. This is comparatively early. *P. massiliensis *starts feeding only at the 10-chaetigerous [52] or 13-chaetigerous [43] segments stage respectively when it leaves the tube of the mother.

Another developmental difference between nereidids that can be attributed to the presence or absence of a pelagic larval stage is found in the "egg jelly". The formation of a jelly-like mass surrounding the eggs is the first indicator of a successful fertilization in *P. dumerilii*, resulting from the cortical reaction of the zygotes after fertilization [5,6,40]. In *P. dumerilii *the jelly has several functions: 1) it blocks fusion of supernumerary sperm, 2) it shelters the egg and 3) it reduces sinking and enhances floating of the eggs [6]. In *P. massiliensis *only a thin jelly layer is formed, which corresponds to Gilpin-Brown's [49] observation that a thick jelly envelope is a typical feature of nereidids with pelagic larvae.

### Loss of developmental synchrony during larval settlement

The development of *P. dumerilii *is highly synchronized only until the late nectochaete stage, at which point synchrony is lost and the larvae develop at very different individual rates [6]. This relates to larval settlement and to the onset of feeding (which can vary from five [6] to ten days after fertilization [47]). As a consequence, siblings from the same batch, which live in the same box in culture [6], can show enormous differences in the number of segments.

The loss of synchrony at later stages is also described for other nereidid species. For example, Hempelmann [52] finds variable developmental rates for *P. massiliensis*, and Gilpin-Brown [49] mentions that the growth rate becomes more variable in the three-chaetigerous larvae in *N. fucata*.

Various environmental parameters relevant for larval settlement appear to influence the rate of development. It has been demonstrated for *P. dumerilii *that seawater conditioned with starving adults for 24 hours slows down the development of the larvae remarkably [79]. Sato and Tsuchiya [80] showed that the development of *Hediste atoka *(previously called *Nereis atoka*) is slower at a salinity of 23‰ compared to 15‰. Also, Smith [81] shows that a salinity/chlorinity of 5 g Cl/L or 15 g Cl/L leads to developmental retardation in *Hediste diversicolor *(previously called *Nereis diversicolor*) compared to 10 g Cl/L in the control. It is possible that other parameters like the oxygen content, light or different genetic background affect developmental pace as well.

### Temperature control of developmental speed

The comparison of *P. dumerilii *larvae raised at different temperatures shows that even a small difference in temperature results in an enormous difference of the developmental stage reached at a given time post fertilization and the developmental pace correlates strongly with the water temperature. *P. dumerilii *eggs and larvae can develop within a wide temperature range between at least 14 and 30°C. The comparison of the developmental stages shows that an increase of the temperature about 10°C from 18 to 28°C leads to a more than two-fold increase of the developmental pace. Such an increase would be expected from the temperature dependence of enzymatic activities according to the Arrhenius equation, which states that an increase of the temperature by 10°C leads to a 2-to-4-fold increase of the reaction efficiency. A similar correlation between water temperature and developmental pace was also reported for e.g. *Scolecolepides viridis *[82].

A general dependence of developmental speed on temperature has previously been reported for other polychaetes such as *Lepidonotus *sp. [83] or *Polydora giardi *[74]. Wilson [42] emphasizes that the temperature dependence of the developmental speed allowed him, by cooling down subsets of larvae, to study the same process in specimens from the same batch several times. Gilpin-Brown [49] points out that in *N. fucata *the speed of development is highly variable - most likely determined by differences in temperature.

## Conclusions

We present the first comprehensive atlas and staging system of *Platynereis dumerilii *normal development. An overview of all stages including schematic drawings for each stage covering the most important morphological characteristics is given in Figure 1, 2, 3, 4. Stage names have been adopted, whenever feasible, from commonly used terminology for annelid larvae [6,36,62]. The atlas includes light microscopy images for reference as well as confocal scans of the stage-specific nervous system and musculature.

## Methods

### *Platynereis dumerilii *culture

*P. dumerilii *larvae were obtained from an established breeding culture, following Dorresteijn *et al. *[12], and were raised in a climate chamber at 18°C ± 0.1°C (Type KB53, Binder, Tuttlingen, Germany). To test the effect of different temperatures on the developmental rate some larvae were kept at 9°C ± 0.5°C, 14°C ± 0.1°C, 16°C ± 0.5°C, 20°C ± 0.5°C, 25°C ± 0.5°C, 28°C ± 0.5°C, 30°C ± 0.5°C and 34°C ± 0.5°C.

### Sampling, Fixation and Staining

In total 15 batches were split into around six parts each and fixed at different time points. For each stage two to five samples were taken and analyzed. Furthermore, the author A.H.L.F. worked with *P. dumerilii *for over three years handling in average three to five batches at different developmental stages per week, which enables the authors to recognize normally developing larvae.

Larvae were fixed in 4%PFA in PBS + 0.1% Tween-20 (PBT), for 50 min at room temperature, rinsed in PBT 2 × 20 min and stored in PBT at 4°C for up to 7 days. The larvae were Proteinase K-digested and post-fixed as described in Tessmar-Raible *et al. *[15]. Specimens and antibodies were blocked in 5% sheep serum in PBT and incubated over one to three nights shaking at 4°C in the primary antibodies mouse anti acetylated alpha-tubulin (Sigma T6793) and rabbit anti 5-HT (serotonin) (DIASORIN, #13002307) 1:500 dilution. Before incubating the larvae in the secondary antibody, the specimens were washed 3 × 10 min and 3 to 5 × 30 min in PTW and the larvae and antibodies were blocked 1 h in 5% sheep serum in PBT. The larvae were incubated 1-3 nights shaking at 4°C in anti mouse FITC (Jackson ImmunoResearch) 1:250, rhodamine phalloidin (Molecular Probes) 1:100, anti rabbit Cy5 (Jackson ImmunoResearch) 1:250 and DAPI (1 μg/μl final concentration). Following antibody incubations, the larvae were washed as described above and stored in 87% glycerol containing 2.5 mg/mL of anti-photobleaching reagent DABCO (Sigma, St. Louis, MO, USA) at 4°C. Incubation of the larvae in secondary antibodies without prior incubation in primary antibodies does not result in any staining (data not shown).

### Microscopy

Fixed and living larvae were mounted between a slide and a cover slip, separated by two to five layers of adhesive tape.

All bright-field images were taken from living specimens, just after collecting them at 18°C ± 0.1°C. Bright-field images were taken on a Zeiss Axiophot microscope using DIC optics. Larvae from mid-trochophore stage onwards show muscle contractions. They were anesthetized in a 1:1 mixture of natural seawater and a 7.5% (w/v) MgCl_2 _solution (described in Ackermann *et al. *[20]) in order to take bright-field images. Stacks of bright-field images were merged into single images by the software Helicon focus and processed further with Photoshop to enhance contrast, rotate and crop the images.

Confocal images were taken on a Leica TCS SPE with a 40× oil immersion objective using appropriate laser lines. For each larva, 60-210 1 μm thick sections were taken and processed with Imaris, ImageJ and Photoshop.

## Abbreviations list

1com: first commissure; 1to: primary tooth; 2com: second commissure; 2to: secondary tooth; 3com: third commissure; 48CS': 48^th ^chaetigerous segment after cephalic metamorphosis; 49CS': 49^th ^chaetigerous segment after cephalic metamorphosis; 4CS: 4^th ^chaetigerous segment; 5CS: 5^th ^chaetigerous segment; 4CS': 4^th ^chaetigerous segment after cephalic metamorphosis; 5CS': 5^th ^chaetigerous segment after cephalic metamorphosis; 6CS': 6^th ^chaetigerous segment after cephalic metamorphosis; 7CS': 7^th ^chaetigerous segment after cephalic metamorphosis; ac: anal cirrus; act: akrotroch; adc: anterior dorsal cirrus; ae: adult eyes; an: antennal nerve; ant: antenna; antm: antennal muscle; apt: apical tuft; avc: anterior ventral cirrus; cc: cerebral commissure; ch: chaetae; chs: chaetal sac; com: commissure; con: connective; CLSM: Confocal laser scanning microscopy; dbv: dorsal blood vessel; dcpp: dorsal cirrus of the parapodia; dlm: dorsal longitudinal muscle; drcc: dorsal root of the circumesophageal connectives; fp: food particles; gz: growth zone; hpf: hours post fertilization; j: jaw; ld : lipid droplet; le: larval eye; mg: midgut; mm: macromere; mt: metatroch; mvlm: median ventral longitudinal muscle; om: oblique muscle; ooc: oocyte; pat1: first paratroch; pat2: second paratroch; pdc: posterior dorsal cirrus; pi: pigment; pl: palpus; pln: palpus nerve; pp: parapodia; ppm: parapodial muscle; pro: proctodeum; pt: prototroch; ptrn: prototroch ring nerve; pvc: posterior ventral cirrus; RNAi: RNA-interference; sf: stomodeal field; sn: segmental nerve; stm: musculature around the stomodeum; sto: stomodeum; str: stomodeal rosette; th: teeth; tt: telotroch; ua: unpaired dorsal axon; veg: vegetal pole; vlm: ventral longitudinal muscle; vrcc: ventral root of the circumesophageal connectives

## Competing interests

The authors declare that they have no competing interests.

## Authors' contributions

AHLF conceived the study and wrote the first draft of the manuscript, conducted the fluorescent stainings, the CLSM analysis, the 3 D reconstruction and the light microscopy, TH generated the online database and the stage ontology in OBO format, DA contributed substantially to the interpretation of data and to the writing of the manuscript. All authors read and approved the final manuscript.
